# Integrated Multiregional Analysis Proposing a New Model of Colorectal Cancer Evolution

**DOI:** 10.1371/journal.pgen.1005778

**Published:** 2016-02-18

**Authors:** Ryutaro Uchi, Yusuke Takahashi, Atsushi Niida, Teppei Shimamura, Hidenari Hirata, Keishi Sugimachi, Genta Sawada, Takeshi Iwaya, Junji Kurashige, Yoshiaki Shinden, Tomohiro Iguchi, Hidetoshi Eguchi, Kenichi Chiba, Yuichi Shiraishi, Genta Nagae, Kenichi Yoshida, Yasunobu Nagata, Hiroshi Haeno, Hirofumi Yamamoto, Hideshi Ishii, Yuichiro Doki, Hisae Iinuma, Shin Sasaki, Satoshi Nagayama, Kazutaka Yamada, Shinichi Yachida, Mamoru Kato, Tatsuhiro Shibata, Eiji Oki, Hiroshi Saeki, Ken Shirabe, Yoshinao Oda, Yoshihiko Maehara, Shizuo Komune, Masaki Mori, Yutaka Suzuki, Ken Yamamoto, Hiroyuki Aburatani, Seishi Ogawa, Satoru Miyano, Koshi Mimori

**Affiliations:** 1 Department of Surgery, Kyushu University Beppu Hospital, Beppu, Japan; 2 Department of Otorhinolaryngology, Graduate School of Medical Sciences, Kyushu University, Fukuoka, Japan; 3 Department of Gastroenterological Surgery, Graduate School of Medicine, Osaka University, Suita, Japan; 4 Laboratory of DNA Information Analysis, Human Genome Center, Institute of Medical Science, University of Tokyo, Tokyo, Japan; 5 Department of Surgery, Iwate Medical University, Morioka, Japan; 6 Genome Science Laboratory, Research Center for Advanced Science and Technology, University of Tokyo, Tokyo, Japan; 7 Department of Biology, Faculty of Sciences, Kyushu University, Fukuoka, Japan; 8 Department of Pathology and Tumor Biology, Kyoto University, Kyoto, Japan; 9 Department of Surgery, Teikyo University School of Medicine, Tokyo, Japan; 10 Department of Surgery, Omori Red Cross Hospital, Tokyo, Japan; 11 Gastroenterological Center, Department of Gastroenterological Surgery, Cancer Institute Hospital, Japanese Foundation for Cancer Research, Tokyo, Japan; 12 Department of Surgery, Takano Hospital, Kumamoto, Japan; 13 Division of Cancer Genomics, National Cancer Center Research Institute, Tokyo, Japan; 14 Department of Surgery and Science, Graduate School of Medical Sciences, Kyushu University, Fukuoka, Japan; 15 Department of Anatomical Pathology, Graduate School of Medical Sciences, Kyushu University, Fukuoka, Japan; 16 Medical Genome Sciences, Graduate School of Frontier Sciences, University of Tokyo, Kashiwa-shi, Chiba, Japan; 17 Department of Medical Chemistry, Kurume University School of Medicine, Kurume, Japan; Institute of Cancer Research, UNITED KINGDOM

## Abstract

Understanding intratumor heterogeneity is clinically important because it could cause therapeutic failure by fostering evolutionary adaptation. To this end, we profiled the genome and epigenome in multiple regions within each of nine colorectal tumors. Extensive intertumor heterogeneity is observed, from which we inferred the evolutionary history of the tumors. First, clonally shared alterations appeared, in which C>T transitions at CpG site and CpG island hypermethylation were relatively enriched. Correlation between mutation counts and patients’ ages suggests that the early-acquired alterations resulted from aging. In the late phase, a parental clone was branched into numerous subclones. Known driver alterations were observed frequently in the early-acquired alterations, but rarely in the late-acquired alterations. Consistently, our computational simulation of the branching evolution suggests that extensive intratumor heterogeneity could be generated by neutral evolution. Collectively, we propose a new model of colorectal cancer evolution, which is useful for understanding and confronting this heterogeneous disease.

## Author Summary

Cancer is heterogeneous disease; each tumor in different patients has different cancer genomes. Furthermore, another level of heterogeneity exists: even a single tumor harbors multiple genetically distinct subclones. This intratumor heterogeneity is presumably one of causes of therapeutic difficulty, and its understanding is clinically necessary. In this study, we investigated intratumor heterogeneity in colorectal cancer by analyzing sample obtained from geographically separated regions of 9 colorectal tumors. Our integrated data analyses combined with computational simulation strongly suggest that, after clonally shared alterations were accumulated by aging, numerous subclones were generated by neutral evolution. Importantly, this view can explain the robustness and evolvability of cancer: therapeutic action inducing an environmental change would convert some of the numerous neutral mutations to driver genes that confer therapeutic resistance. We believe that this study not only provides insights into colorectal cancer pathogenesis, but also constitutes a new basis for designing therapeutic strategies.

## Introduction

Cancer is a heterogeneous disease. Recent cancer genomics studies have revealed extensive genetic diversity among patients. Moreover, even a clonal tumor in one patient often harbors multiple subclones. This phenomenon is called intratumor heterogeneity (ITH) and is presumably generated by branching clonal evolution of cancer cells. Understanding of ITH is clinically important, since the existence of multiple subclones presumably boosts the evolutionary adaptation of tumors against therapies, constituting a source of resistant clones [1].

Recently, a multiregional sequencing approach, which sequences DNA sampled from geographically separated regions of a single tumor, has revealed branched evolution and ITH. Yachida et al. [2] investigated the genomic evolution of pancreatic cancer, establishing two categories of mutations: “founder” and “progressor” mutations are present in all regions and a subset of regions, respectively. Founder mutations are assumed to appear in the early phase of clonal evolution. We refer to the clone that has accumulated all the founder mutations as the parental clone (or the most recent common ancestor). The parental clone then branches into subclones by accumulating progressor mutations, which shape ITH.

Several studies employing multiregional exome sequencing have revealed the occurrence of branched evolution and ITH in several other types of cancers, including clear cell renal cell carcinomas and non-small cell lung cancers. ITH of clear cell renal cell carcinomas is characterized by parallel evolution, in which the same driver gene is independently mutated in different branches of evolutionary trees [3]. In contrast, no evidence of parallel evolution has been reported for non-small cell lung cancer [4, 5]. In addition to genetic aberrations, epigenetic aberrations are also a hallmark of cancer; as for DNA methylation, a few groups have also performed multiregional epigenomic analyses [6, 7]. However, the types of cancers that have been subjected to multiregional analyses remain limited, and ITH of genomes and epigenomes has been poorly studied in an integrated way.

In this study, we present genetic and epigenetic analysis of ITH in a series of nine colorectal cancers. Following multiregional sampling, we performed exome sequencing and copy number (CN), methylation, and mRNA expression array profiling. Our integrated analysis revealed not only extensive ITH, but also the evolutionary histories of the nine tumors. Finally, we also performed computational simulation of cancer evolution, which suggested a possible evolutionary principle underlying the extensive ITH.

## Results

### Multiregional exome sequencing unveils extensive ITH and branched evolution

**Fig 1:**
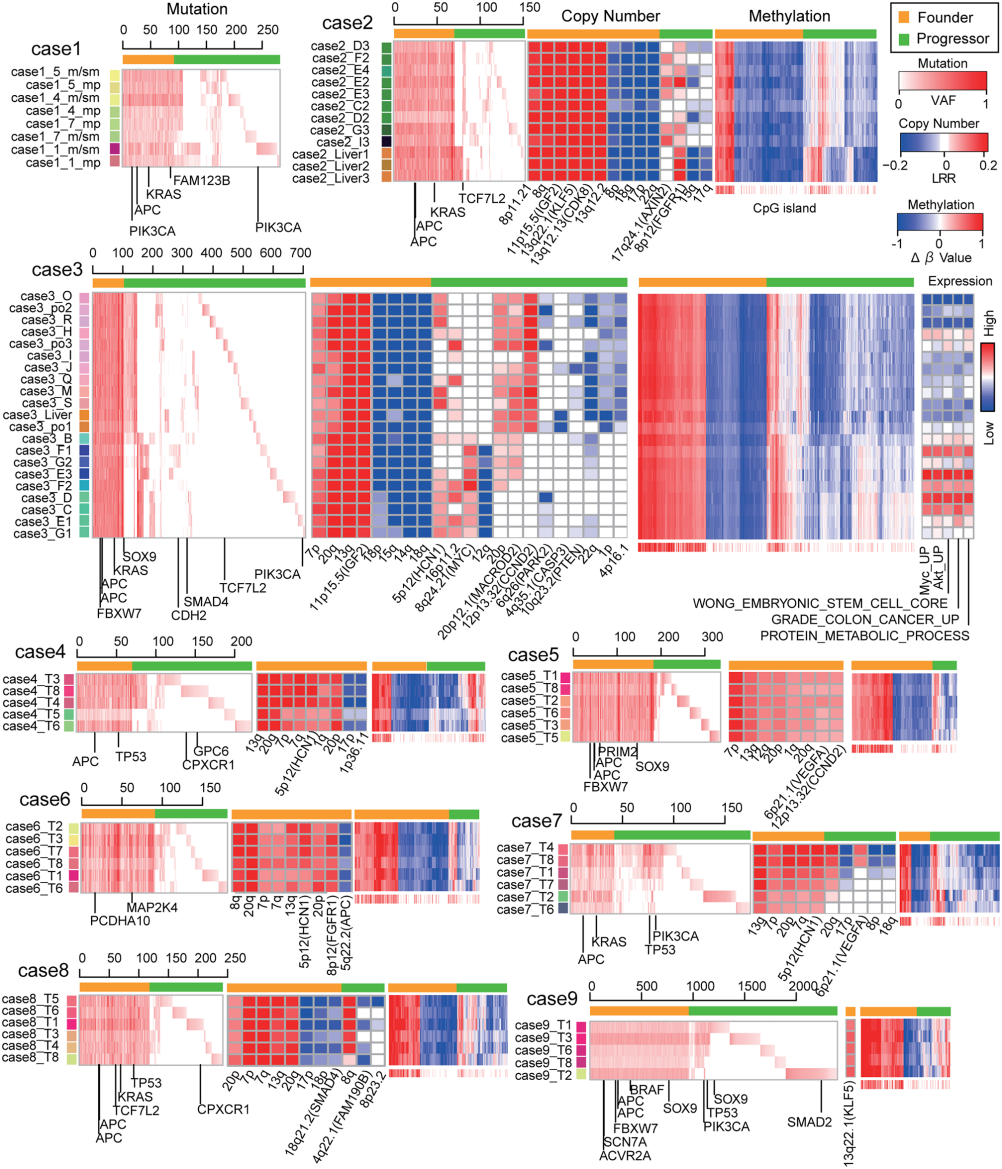
An integrated view of ITH in the 9 colorectal tumors. Multiregional profiles of mutations, CN and methylation alterations were visualized as heat maps. Orange and green bars indicated founder and progressor alterations, respectively. Colored labels for each sample were prepared so that color similarity represents similarity between mutation profiles. For case3, activities of expression signatures were also provided.

To study ITH in colorectal cancer, we performed genomic analysis of samples from geographically separated regions from nine colorectal tumors (S1 Table). In this study, we referred to the nine patients by the term “case” and to multiregional samples in each case by the term “sample”. From each of the nine tumor, we obtained 5–21 multiregional samples, which were 75 samples in total, together with 9 paired normal mucosa samples (S2 Table). For two cases, samples from liver metastases were obtained. Our multiregional exome sequencing of the nine cases found 16857 mutations in total, for an average of 58–1195 mutations per sample (S3 Table). From these values, the mutation rates for each case were estimated to be 1.57–20.2 mutations per megabase. All cases, except for case 9, fall in a range typical for non-hypermutated colorectal cancer [8]. Mutational profiles obtained from the multiregional sequencing demonstrated high genetic ITH for all nine colorectal tumors (Fig 1).

**Fig 2:**
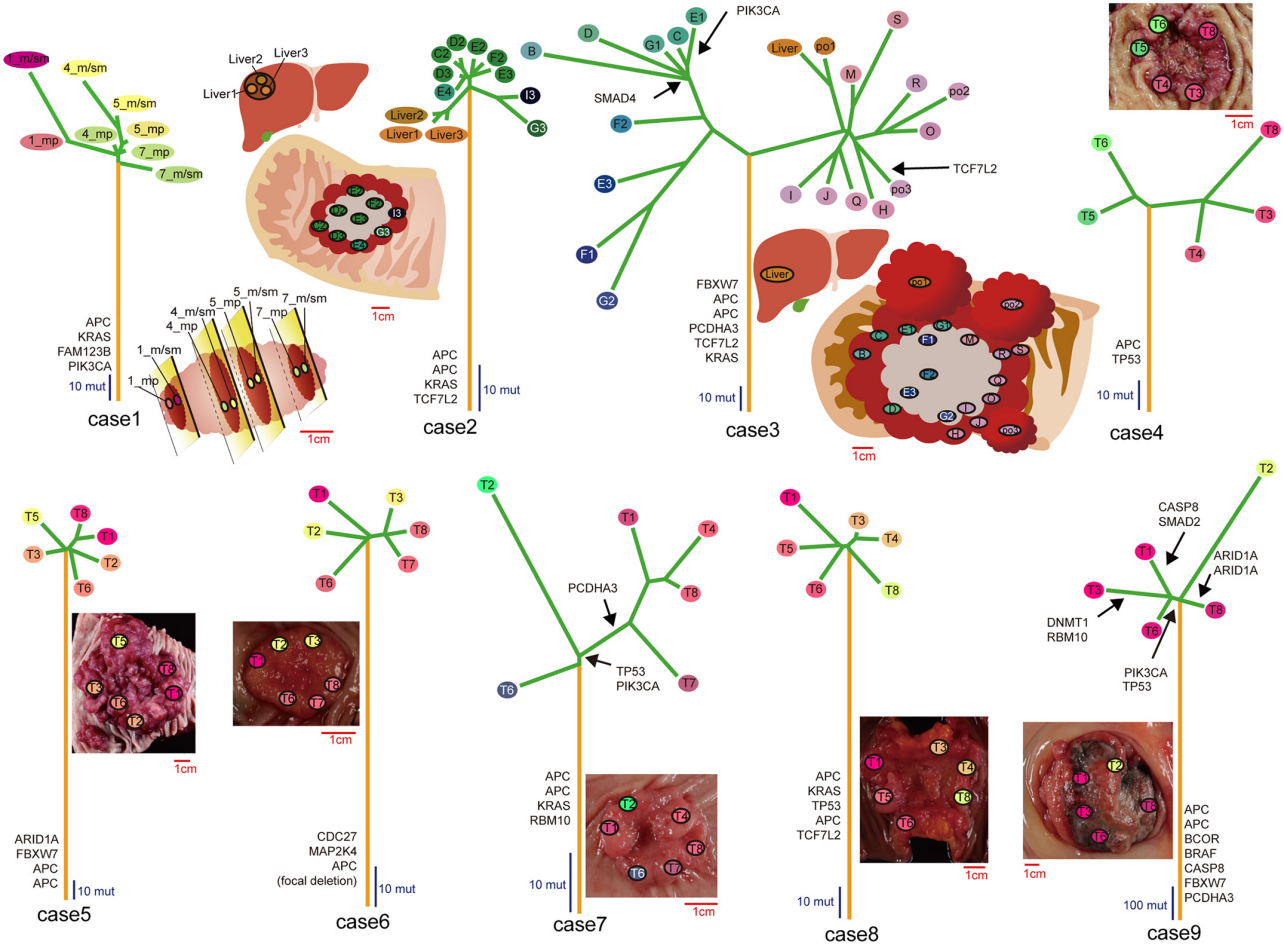
Evolutionary trees of the 9 colorectal tumors. Evolutionary trees inferred from the multiregional mutation profiles have orange trunks, green branches and variously colored leaves, which correspond to founder, progressor mutations and samples, respectively. The leaves were colored based on the color-coding scheme used in Fig 1. Mutation timings of reported driver genes in colorectal cancer were indicated along the trees, and schemas or photos of multiregionally sampled tumors were also provided. Red and blue scales measure tumor size and tree size based on the number of mutations, respectively.

Each of the multiregional mutation profiles harbored founder and progressor mutations; founder mutations are shared by all regions while progressor mutations are not. We further divided progressor mutations into two subcategories: “unique” and “shared” mutations, which are unique to a single specific sample and shared by multiple but not all samples, respectively. Targeted deep sequencing validated 100% (5068/5068), 93.9% (1745/1857) and 95.4% (1362/1427) of founder, shared, and unique mutations, respectively. We can assume that founder, shared, and unique mutations are acquired in this order during cancer evolution. Applying the maximum parsimony method [9] to the multiregional mutation profiles allowed us to depict the evolutionary trees of the nine tumors (Fig 2). Comparison between the evolutionary trees and geographical positions of each of the samples showed that subclones were generally separated in geographically correlated ways, demonstrating that geographical relations are maintained as the evolution of colorectal cancer proceeds. On the other hand, our analysis of the deep sequencing data revealed that some regions in two cases harbor intermixed subclones from separated regions, which confirmed a recent finding by Sottoriva et al. (S2 Fig) [10].

**Fig 3:**
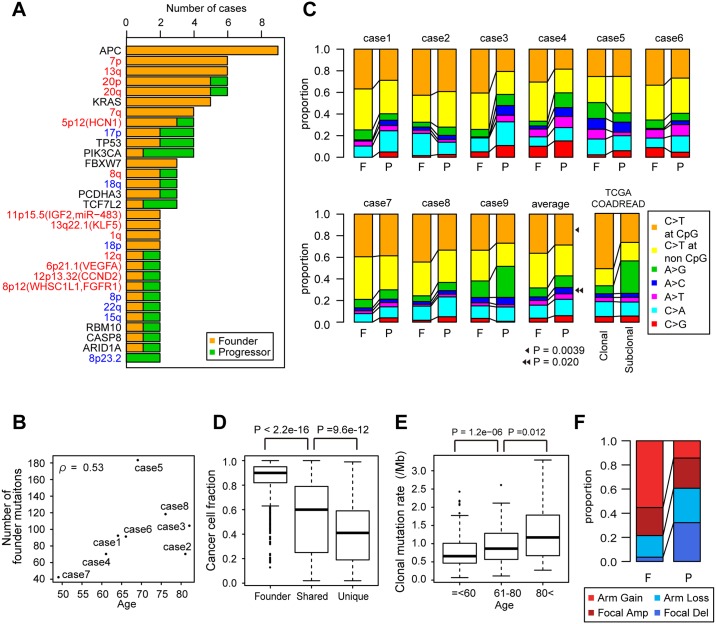
Analysis of genetic ITH. (**A**) The number of samples having mutation (black letters), CN gains (red letters) and losses (blues letters) were counted for each of the founder and progressor categories. The APC item counts one sample subjected to focal deletion. (**B**) Correlation between founder mutation counts and patients’ ages in our 8 cases. Case 9 was excluded due to a hypermutation phenotype. ρ is Spearman’s correlation coefficients. (**C**) Mutational signatures were calculated from founder (F) and progressor (P) mutations in our 9 cases, and also from clonal and subclonal mutations in non-hypermutated TCGA samples. P-values were calculated by Wilcoxon signed-rank test on the 9 cases. (**D**) Distribution of cancer cell fraction in which founder, shared and unique mutations occur. P-values were calculated by The Wilcoxon rank-sum test. (**E**) Correlation between clonal mutation rates and patients’ ages in TCGA non-hypermutated samples. P-values were calculated by The Wilcoxon rank-sum test. (**F**) Proportion of arm-level gain, loss, focal amplification and deletion was calculated for founder and progressor CN alterations in the 8 cases subjected to CN profiling.

We found that mutations in well-known driver genes such as *APC*, *KRAS*, and *FBWX7* were acquired as founder mutations during the establishment of the parental clones (Fig 3A). Pathway-level analysis also showed that founder mutations disrupted the WNT and RTK/RAS pathways, consistently with their principal roles in colorectal tumorigenesis (S3 Fig). Once the parental clones were established, these clones branched into subclones by accumulating progressor mutations. We found that mutations in *PIK3CA* recurrently occurred as progressor mutations, suggesting that *PIK3CA* mutations are a late event in the evolution of colorectal cancer. On the other hand, we did not find any evidence of parallel evolution, as has been observed in studies of clear cell renal cell carcinomas [3].

### Analysis of genetic ITH suggests that early-acquired mutations results from aging

We counted each category of mutation and then identified a correlation between the number of founder mutations and the age of the patients (Fig 3B, S4 and S5 Figs). Our findings are consistent with the model of founder mutations accumulating from aging, and similar correlations have also been observed in other types of solid tumors, such as pancreatic cancer and clear cell renal cell carcinomas (S5 Fig). To investigate the temporal signatures embedded in the mutations, we then compared mutational signatures between founder mutations and progressor mutations (Fig 3C). Our analysis showed that C > T transitions at CpG sites are more prominent in founder mutations than in progressor mutations. Next, we calculated the fraction of cancer cells harboring each category of mutation from variant allele frequency (VAF), read depth and CN data (Fig 3D). We found that the cancer cell fractions decreased while proceeding from founder to shared and unique mutations; that is, founder and progressor mutations tend to exist as clonal and subclonal mutations in each sample, respectively. In our multiregional sampling, we estimated that the cell population sizes of each sample are about 10^6^ from the amount of DNA, while those of each case as a whole are from 10^9^ to 10^10^ based on tumor size. This observation suggests that ITH is extremely extensive and the resolution of our multiregional sampling remains insufficient to reveal its totality.

This result also prompted us to estimate founder and progressor mutations in single sample sequencing data (S6 Fig); from The Cancer Genome Atlas (TCGA) colon and rectum adenocarcinoma exome sequencing data [8], we obtained clonal and subclonal mutations as surrogates of founder and progressor mutations, respectively. Using this data, we confirmed that clonal mutations are increased with patients’ ages and that they have a higher proportion of C>T transitions at CpG sites than do subclonal mutations (Fig 3C and 3E and S7 Fig). A recent pan-cancer analysis reported that C>T transitions at CpG sites are positively correlated with patients’ ages [11]. Consistently, we confirmed that C>T transitions at CpG sites are increased with patients’ ages in the TCGA data (S7 Fig). Taken together, the TCGA data analysis supported the hypothesis that founder mutations enriched with C>T transitions at CpG sites were accumulated during aging.

In addition to the exome sequencing, we also performed single-nucleotide polymorphism (SNP) array-based CN profiling for all cases except case 1 (Fig 1 and S9 Fig). The multiregional CN profiles showed that amplifications of 7p, 13q, 10q, 20p, and 20q frequently occurred across all samples in multiple tumors, namely as founder CN alterations (Fig 3A). We also found that, compared with the degrees of mutational ITH, those of CN ITH were variable among cases. For example, cases 2, 3, and 7 showed relatively high CN ITH, which was acquired in a manner that correlated with mutational ITH, as shown by cluster analysis (S9 Fig). In addition to founder CN alterations, we identified CN alterations that occurred along the mutation-based evolutionary trees as progressor CN alterations. We found that arm-level gains tended to occur in founder CN alterations, while focal deletions tended to occur as progressor CN alterations (Fig 3F).

### Analysis of epigenetic ITH shows that CpG island hypermethylation occurs early in the evolution

**Fig 4:**
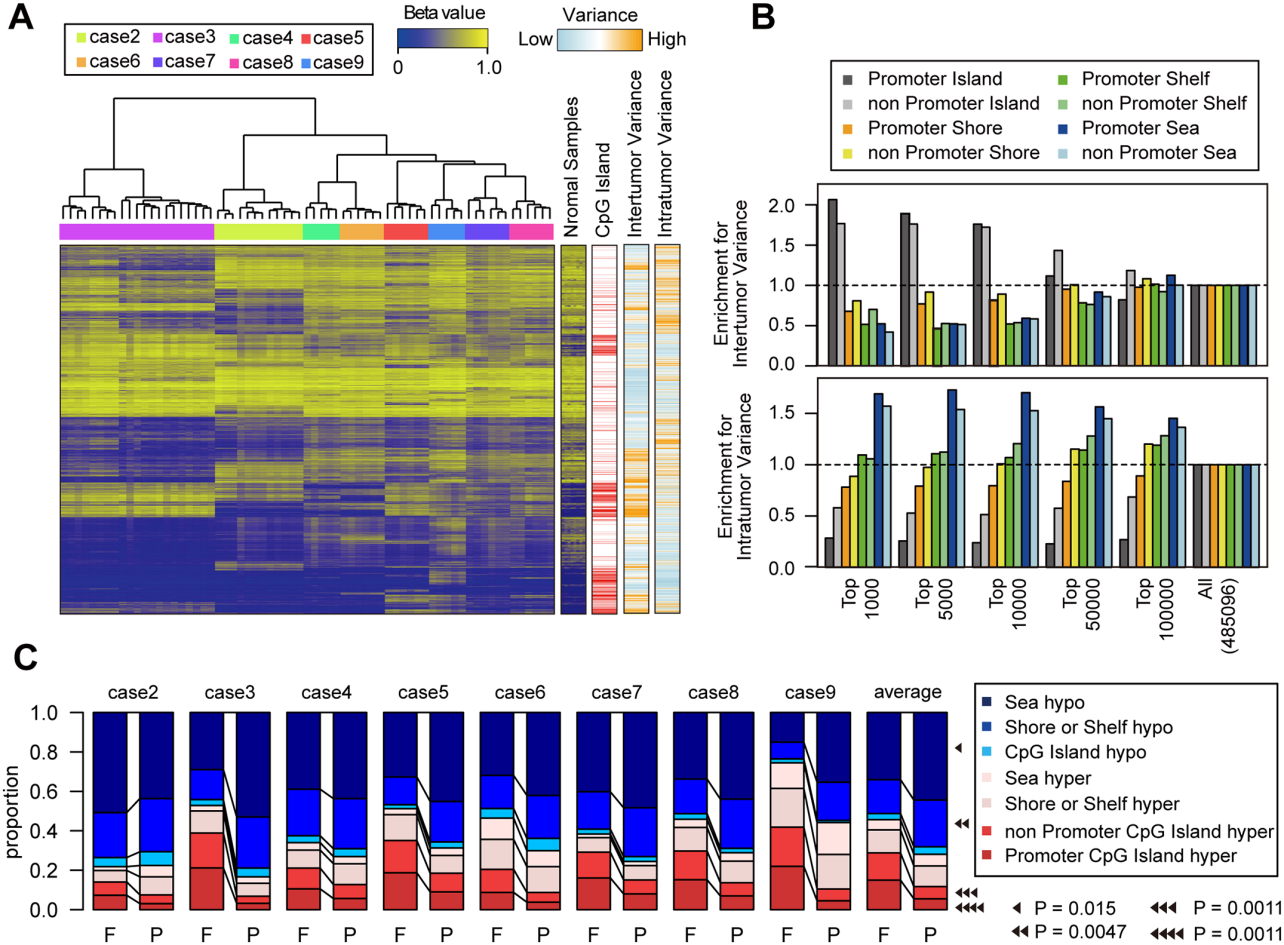
Analysis of epigenetic ITH. (**A**) A heat map of multiregional methylation profiles of the 8 cases. (**B**) Differential contribution of different categories of probe to intratumor and intertumor variance. According to intratumor and intertumor variance, Probes were ranked to obtain each indicated number of top-ranked probes. Probes were categorized based on their genomic positions and enrichment of each category in the top-ranked probes was measured. (**C**) Proportion of indicated types of methylation alterations was calculated for founder (F) and progressor (P) methylation alterations in the 8 cases. P-values were calculated by Wilcoxon signed-rank test on the 8 cases.

To examine epigenetic ITH, we obtained DNA methylation array data for eight cases. Cluster analysis of the multiregional methylation profiles revealed tight clustering of each case, indicating that intertumor heterogeneity (i.e., heterogeneity among cases) is dominant over ITH (Fig 4A). However, we did observe substantial ITH for each case and, to analyze epigenetic ITH, we focused on variance in methylation levels of each probe in multiregional methylation profiles. Note that the total variance can be decomposed into variance among cases and within cases (hereafter referred as to inter- and intratumor variance, respectively). Notably, we found that different categories of methylation probes contribute differently to the two types of variance (Fig 4B, S10 Fig). We categorized probes based on positional information: relative distances to CpG islands and whether the probes are located in promoter regions. We found that probes within CpG islands tended to show higher intertumor variance than those outside CpG islands. This is consistent with the fact that CpG island hypermethylation marks epigenetic subtypes in colorectal cancer [12]; in our study, cases 5 and 9 appeared to fall into the CpG island methylator phenotype (CIMP) subtype as shown by cluster analysis combined with TCGA samples (S11 Fig). On the other hand, methylation probes outside CpG islands tended to show higher intratumor variance than those within CpG islands. This observation possibly reflects the unstable nature of methylations outside CpG islands, and indicates that methylation alterations outside CpG islands are a main contributor to epigenetic ITH. We also performed similar analysis using classification of chromosomal regions based epigenetic status in normal colon tissue (S12 Fig)[13]. We found that the bivalent domains, which are marked by the H3 lysine 4 and H3 lysine 27 methylation and reportedly involved in cellar differentiation, were associated with high intertumor variance. On the other hand, no clear association existed between any specific region category and intratumor variance, suggesting no functionality of epigenetic ITH.

To further investigate ITH and evolution in the colorectal cancer epigenomes, we identified founder and progressor methylations, as done for genetic alterations (S13 Fig). We also differentiated hyper- and hypomethylations by reference to methylation levels in paired normal mucosa samples. Founder methylations are defined as hyper- or hypomethylations that commonly occur across all samples within each case. As expected, we found that the loss of epigenetic gatekeepers such as SFRPs, GATA4, and GATA5 [14] is incurred by founder hypermethylation in many cases (S14 Fig). We also identified hyper- or hypomethylations that occurred along the mutation-based evolutionary trees as progressor methylations. We then deduced temporal signatures from the multiregional methylation data by combining the three types of categorizations: founder and progressor methylation; hyper- and hypomethylation; and positional categories of methylation probes (Fig 4C). Our data showed that hypermethylations in CpG islands were more prominent in founder methylations than in progressor methylations, suggesting that CpG island hypermethylations mainly occur in the early phase of colorectal cancer evolution. Intriguingly, connections between epigenetic aberration and aging have been reported [15], and our analysis of TCGA data also demonstrated that patients’ ages were correlated with the number of hypermethylated probes, but not with that of hypomethylated probes (S15 Fig). Collectively, our results suggest that CpG island hypermethylations, as early events in the evolution of colorectal cancer, result from aging, consistent with our findings regarding mutations. On the other hand, enrichment of hypomethylations in progressor methylations suggests that global hypomethylation starts at a relatively late stage in the evolution, and shapes a substantial part of epigenetic ITH.

### An integrated view of ITH tells a cancer’s life history

By combining mutation, CN, and methylation profiles, we obtained integrated views of ITH in a series of colorectal cancer samples (Fig 1). For case 3, we additionally obtained mRNA expression profiles. From these integrated views, as well as from evolutionary trees, we can envision the life history of each tumor. Here, we describe that of case 3 as an example. In the founder phase, the parental clone accumulated founder mutations together with CN gain and loss and hyper- and hypomethylation. The founder mutation contains driver mutations represented by mutations in *APC*, *KRAS*, and *FBWX7*. In the progressor phase, the parental clone divided into two major subclones. One major subclone had focal *MYC* amplification, suggesting that this major subclone was shaped by positive natural selection. Although not having a clear driver alteration, the other major subclone had several shared CN alterations, such as 20p amplification and 1p deletion. Then, each of the two major subclones branched into minor subclones, while accumulating many progressor alterations. During this process generating ITH, mutation accumulations, CN alterations, and methylation alterations appeared to occur in a correlated manner. We also found ITH in the transcriptome; notably, the major subclone harboring *MYC* amplification showed upregulation of the *MYC* expression signature together with other signatures related to cancer malignancy. The case 3 tumor also contains a sample from liver metastasis, and the evolutionary tree suggests that the liver metastasis occurred late in the evolution, from a polypoid-like part containing the po1 sample. The metastatic sample of case 3 is contained by the major subclone showing a lower activity of the *MYC* expression signature, which is unexpected if we assume that metastasis results from the acquisition of a malignant phenotype during the evolution. In case 2, which also contains liver metastatic samples, the metastatic samples branched out early in the evolution. Although we need more cases to form a general rule regarding metastasis, our data demonstrate that the multiregional approach is effective to obtain information about the manner in which metastatic clones evolve.

### Simulation of cancer evolution suggests that extensive ITH is generated by neutral evolution

**Fig 5:**
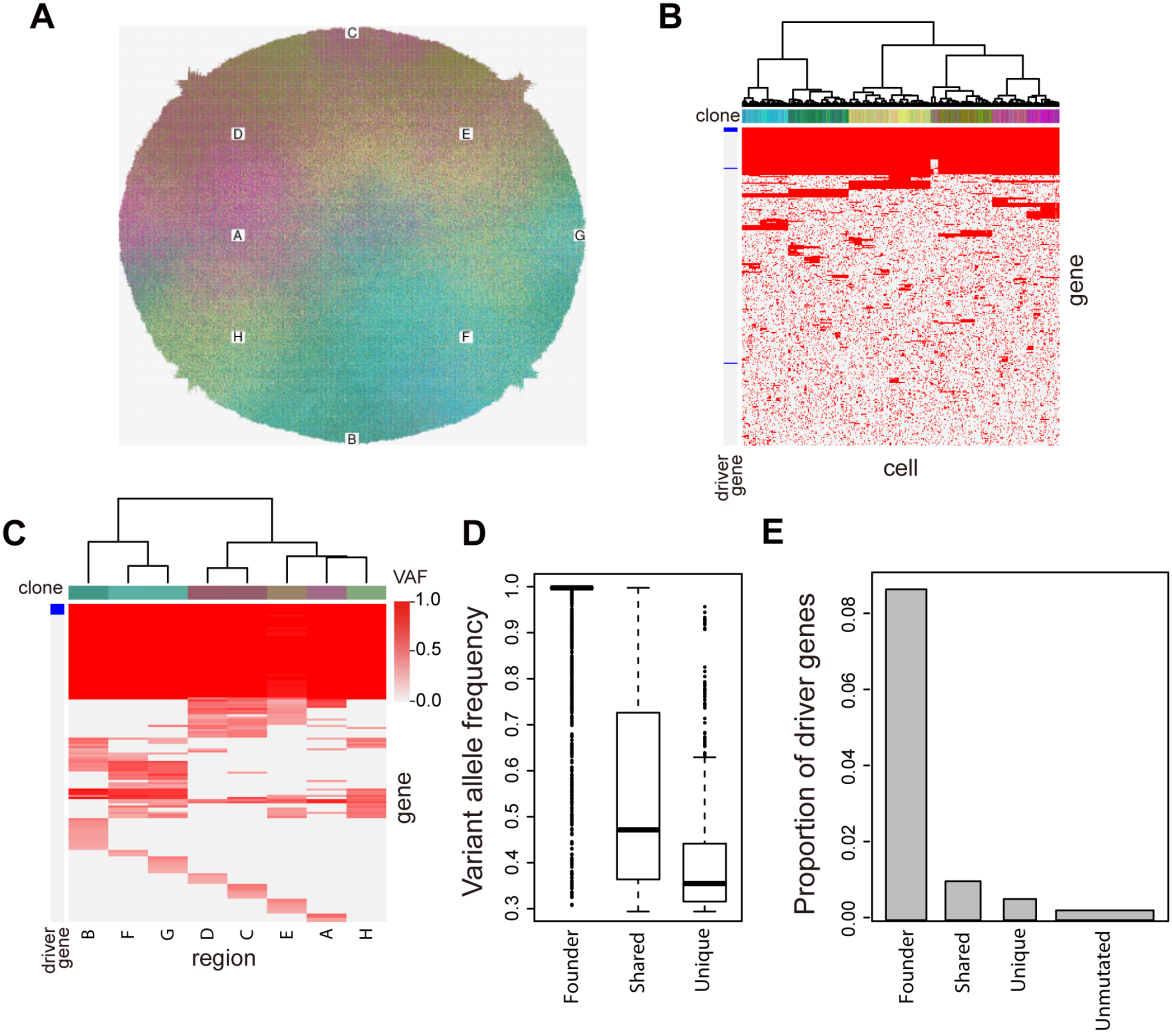
Simulation of cancer evolution. (**A**) A simulated tumor. Different colors represent different clones. White rectangles labeled with alphabets indicate regions subjected to multiregional sampling. (**B**) A simulated single-cell mutation profile matrix. Columns represent 500 cells sampled from the simulated tumor, and the top colored bars label each clone. Rows represent mutated genes and driver genes are indicated by left blue bars. (**C**) A simulated multiregional mutation profile matrix. VAFs of each gene were calculated for cell subpopulations from the 8 regions indicated in (A). (**D** and **E**) Distribution of VAFs (D) and Proportion of driver genes (E) in different categories of mutations. The mutations were obtained from 20 multiregional mutation profile matrices generated by independent simulation trials. In (E), the width of each bar is proportional to the count of each category of mutations. Therefore, the area of each bar is proportional to the count of driver genes that belong to each category of mutations.

As described so far, our genomic analysis revealed a heterogeneous evolution of colorectal cancer. We found that *PIK3CA* mutations and *MYC* amplification occurred in the progressor phase, suggesting that a fraction of ITH is generated by positive natural selection. However, most of the branches in the evolutionary trees had no clear evidence of such positive natural selection, and our clonality analysis of mutations suggests that ITH exists even in each of the multiregional samples (Fig 3D and S6 Fig). To clarify the principle underlying the extensive ITH, we performed computer simulation of a branching evolutionary process (BEP) in cancer evolution (S16 Fig)[16]. In our BEP simulation, cells proliferate while accumulating random mutations in multiple genes. Among the genes, we assume the existence of driver genes whose mutations confer a growth advantage to cells. In the course of tumor growth, cells having mutations in driver genes are evolutionarily selected and, depending on parameter settings, cells accumulate different combinations of mutations to reproduce ITH. Through a parameter fitting analysis, we found that the BEP simulation can generate heterogeneous mutation profiles similar to the real experimental data, if a high mutation rate, a sufficient number and sufficient strength of driver genes are assumed. (S17 and S18 Figs). We simulated tumor evolution with such a parameter setting and then performed multiregional sequencing of the simulated tumor in silico. Similarly to those of our 9 cases, the simulated multiregional mutation profile harbored founder, shared, and unique mutations while the heterogeneity were well correlated with geographical positions (Fig 5A–5C). Moreover, the VAF of each type of mutation tended to decrease while proceeding from founder to shared and unique mutations (Fig 5D). Note that the VAF is equal to the cancer cell fraction in which the mutation occurs, since the simulated tumors have the haploid genome and no contamination of normal cells. Namely, this result reproduced the local ITH within each of the multiregional samples (Fig 3D). Most importantly, our BEP simulation identified a possible evolutionary principle underlying the extensive ITH. The simulated multiregional mutation profile demonstrated that, while founder mutations occurred in most of the driver genes, progressor mutations rarely occurred in driver genes; namely, most of the progressor mutations were neutral mutations that do not confer a growth advantage (Fig 5E). Taken together with our observation that most of the branches in the evolutionary trees lacked apparent driver alteration, our data suggest that most of the ITH is generated by neutral evolution. It should be noted that our BEP simulation could explain an origin of intertumor heterogeneity. For a probabilistic nature of the model, independent simulation trials even with the same parameter setting generated different multiregional mutation profiles, which remind us of the multiregional mutation profiles unique to each of the nine cases (Fig 1 and S19 Fig).

## Discussion

In this study, our integrated multiregional analysis revealed the ITH and evolutionary history of a series of nine colorectal tumors. In particular, by focusing on founder and progressor mutations, we identified clues for decoding the life history of the tumors. For example, we found that founder mutations included established driver mutations such as *APC*, *KRAS*, and *FBWX7*, and their counts correlated with the ages of patients, suggesting that accumulation of alterations in the early phase results from aging.

It is a well-accepted dogma that cancer results from aging [17]. Moreover, associations between somatic mutations and aging have been studied recently. Welch *et al*. [18] found that acute myeloid leukemia (AML) genomes accumulate mutations as a function of age; furthermore, they also confirmed age-dependent mutation accumulation in hematopoietic stem/progenitor cells. Other recent studies report that somatic mosaicism in blood increases in an age-dependent way, and it also has a positive association with cancer risk [19, 20]. Although the association between somatic mutations and aging has been poorly studied in the context of solid tumors, our findings indicate that an association between somatic mutations and patients’ ages exists in colorectal cancer. During aging, a colorectal stem/progenitor cell presumably accumulates somatic mutations, some of which could unfortunately be driver mutations that transform the normal cell to a parental clone. This view is also consistent with a recent report that a high division rate of colorectal stem/progenitor cells well explains a high lifetime risk of colorectal cancer [21].

Through mutational signature analysis, we also found that CpG transitions at CpG sites more frequently occur in founder mutations than in progressor mutations. This mutational signature is related to spontaneous deamination of 5-methyl-cytosine at CpG dinucleotides and is most predominantly observed in various cancer types. A recent pan-cancer analysis [11] and our TCGA data analysis showed that this mutational signature is positively correlated with patients’ ages, which is consistent with our finding that founder mutations marked by this signature increased with patients’ ages. As for DNA methylation, hypermethylation in CpG islands was more prominent in founder methylation than in progressor methylation. We also found that the number of hypermethylated probes is correlated with patients’ ages in TGCA samples. Taken together, we speculate that CpG island hypermethylation incurred by aging also predisposes a colorectal stem/progenitor cell to tumorigenesis in collaboration with somatic mutations.

Thus, genetic and epigenetic alterations are accumulated during aging, and some of them act as driver alterations that transform the normal cell to a parental clone. Once the parental clone is established, it undergoes branched evolution in a geographically consistent way. In addition to ITH of mutations and CN alterations, we found that epigenetic ITH marked by global hypomethylations is prevalent. Our integrated analysis also showed that the genetic and epigenetic ITH are correlated with each other. In contrast to founder alterations, progressor alterations appeared not to have any known driver alterations, with the exception of a few examples such as *PIK3CA* mutation and *MYC* amplification. There also existed no parallel evolution, which is conspicuous in clear cell renal cell carcinomas [3]. Namely, we found little evidence that positive natural selection shaped the extensive ITH, similar to the findings of recent non-small cell lung cancer studies [4, 5]. Moreover, our clonality analysis of mutations suggested that subclones existed even in each of the multiregional samples. It should be noted that such local ITH is consistent with a recent breast cancer study in which single-cell sequencing identified subclonal mutations occurring at low frequencies [22].

In pursuit of the unknown principles generating such extensive ITH, we performed the BEP simulation. Intriguingly, our simulation suggests that neutral evolution can shape extensive ITH as observed in our multiregional mutation profiles. Notably, our simulation also well explained the local ITH within each of the multiregional samples. Although a single-cell mutation profile showed that a simulated tumor actually harbored numerous subclones, snapshots of the simulated evolution suggested that “macroscopic” subclones, which can be captured by the resolution of multiregional sequencing, were generated by genetic drift in the course of the neutral evolution (S20 Fig). A possible mechanism that boosts the neutral mutations is a high mutation rate, as assumed in our simulation. We speculate that genetic instability is incurred and the mutation rate increases before the branched evolution, which is also indicated by the temporal change of mutational signatures. Our computational analysis also suggests that a cancer stem cell hierarchy can boost the neutral evolution [16]. Most importantly, our view that a tumor harbors numerous neutral mutations can explain the robustness and evolvability of cancer. A therapeutic action induces an environmental change, which would convert some of the numerous neutral mutations to driver genes that confer therapeutic resistance. Consistent with this idea, it has recently been reported that resistance to some targeted cancer drugs may result from the outgrowth of preexisting low-frequency subclones [23].

**Fig 6:**
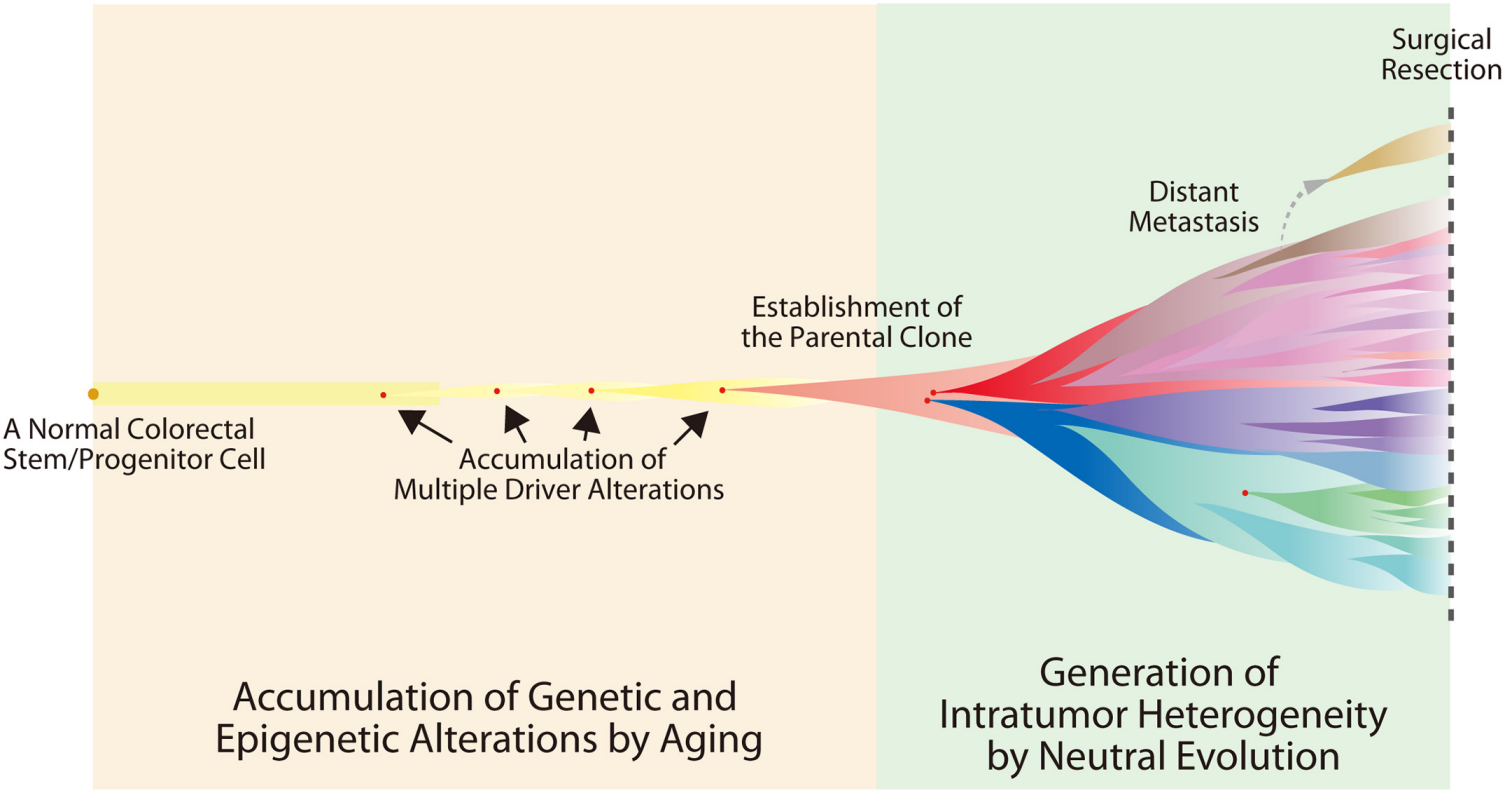
Our model of colorectal cancer evolution. First, founder alterations containing a set of drive alterations are accumulated in the genome and epigenome as a result of aging. After establishment of a parental clone, extensive ITH is generated by neutral evolution, although a few driver alterations are acquired as progressor alterations. Note that this illustration is based on the evolutionary tree of case 3 (Fig 2). However, an actual tumor should harbor numerous subclones, as suggested by the local ITH data (Fig 5D) and simulated single-cell mutation profile (Fig 5B).

Collectively, this work presents a new model of colorectal cancer evolution; aging leads to the accumulation of genetic and epigenetic alterations in the early phase, while neutral evolution shapes extensive ITH in the late phase (Fig 6). Colorectal cancer has been an attractive subject for studying cancer evolution and its evolution have been addressed from various viewpoints [24–28]. Recently, Sottoriva et al. have also proposed that ITH is mainly shaped by neutral evolution, based on uniformly high ITH, subclonal mixing in distant sites and a power-law distribution of VAFs [10, 29]. Along with these works, this study is unique in that it not only unveiled the extensive ITH, but also explained the underlying principle. We believe that our model not only provides insights into colorectal cancer pathogenesis, but also constitute a new basis for designing therapeutic strategies.

## Materials and Methods

### Sample collection and preparation

Nine patients who provided written informed consent were enrolled in this study. Detailed information about participants is provided in S1 Table. The study protocol was reviewed and approved by Kyushu University and cooperative institutes. All samples were obtained during surgery from patients who underwent radical resection of primary and/or liver metastatic tumors at Kyushu University Beppu Hospital, Kyushu University Hospital and Osaka University Hospital. DNA and RNA were extracted from fresh frozen multiregional tumor samples and adjacent normal intestinal mucosa with AllPrep DNA/RNA Mini Kit (Qiagen, Hiden, Germany). For case 1–3, high-purity tumor samples were obtained using a laser microdissection system (Leica Laser Microdissection System; Leica Microsystems, Wetzlar, Germany). A detailed protocol of sample preparation was described previously [30].

### Ethics statement

The study design was approved by the institutional review boards and ethics committees of hospitals that made the practice of patient (Kyushu University Hospital Institutional Review Board: Protocol Number 486–01, Osaka University Institutional Review Board: Protocol Number 12–27). The study was conducted according to the principles expressed in the Declaration of Helsinki. We obtained written informed consent from all the parents in this study. There was no animal experiment in the study.

### Whole exome sequencing (WES)

Whole exome capture was performed with The SureSelct Human All Exon 50Mb kit (Agilent technologies) was used for all samples. The captured targets were subjected to massive sequencing using HiSeq 2000 (Illumina, San Diego, CA, USA) with the pair end 75–100 bp read option. Information of read depth is provided in S1 Fig and S2 Table.

### Mutation calling

The sequence data were processed through an in-house pipeline (http://genomon.hgc.jp/exome/). The sequencing reads were aligned to the NCBI Human Reference Genome Build 37 hg19 with BWA version 0.5.10 using default parameters (http://bio-bwa.sourceforge.net/). PCR duplicate reads were removed with Picard (http://www.picard.sourceforge.net). Mutation calling was performed using the EBcall algorithm [31] with following parameters:

1. Mapping Quality score ≥ 30
2. Base Quality score ≥ 15
3. Both the tumor and normal depths ≥ 8
4. Variant reads in tumors ≥ 4
5. VAFs in tumor samples ≥ 0.08
6. VAF in normal samples ≤ 0.1

### Identification of founder and progressor mutations

For each case, the variants that are called in any samples and whose positions have read depths ≥ 10 in all the samples were obtained and their VAFs in all the samples were calculated by mpileup of samtools-0.1.18. Then, variants whose VAF > 0.05 were finally obtained as somatic mutations. This step rescued mutations that were presumably shared among samples, but missed by EBcall due to disagreement with the above parameter conditions, and also filtered out variants that potentially have false negatives in any samples due to low coverage. This procedure was applied for each case to obtain a multiregional mutation profile, from which we identified mutations shared by all the samples as founder mutations, and others as progressor mutations. Progressor mutations were further divided into shared mutations, which were shared by a subset of samples, and unique mutations, which were unique to a single sample. The mutations were annotated by ANNOVAR (http://www.openbioinformatics.org/annovar/). Information of reported driver genes was based on the TCGA colon and rectum adenocarcinoma (COADREAD) study [8]. Information of all the mutations is provided in S3 Table. The multiregional mutation profile obtained for each case is visualized as a heat map whose intensities represent VAFs. In the heat map, founder mutations were ordered along chromosomal positions, shared mutations were ordered by a hierarchical clustering, and unique mutations were sorted for samples and VAFs. From multiregional mutation profiles, maximum parsimony trees were constructed using the maximum likelihood algorithm in the MEGA6 package [9].

### Color-coding schemes of sample colors

From the multiregional mutation profile of each case, we also deduced a color-coding scheme to prepare color labels of samples. The multiregional mutation profile were regarded as an *n* × *m* matrix, whose *n* columns and *m* rows indexed n mutational positions and m samples, respectively. We applied principle component analysis to the multiregional mutation profile and obtained the first, second and third loading vectors. By multiplying these loading vectors, *n*-dimensional vectors representing mutational profiles of each sample were reduced into three-dimensional vectors. RGB colors used for sample labels are finally papered by mixing red, green and blue proportionally to the three vector elements. In a color-coding scheme deduced by this approach, color similarity reflects similarity of mutation profiles between samples.

### Validation of the mutations by targeted deep sequencing

We validated WES-derived mutations by targeted deep sequencing. Preamplified cDNA library prepared for WES were captured by a custom-designed SureSelect bait library, which included:

1. All progressor mutations in case2-8.
2. At most 100 nonsynonymous mutations randomly selected from founder mutations in each of case2-9.

Enriched targets were sequenced and Sequencing reads were aligned to the NCBI Human Reference Genome Build 37 as done for WES. After the reads that had either mapping quality of <25, base quality of < 30, or ≥ 5 mismatched bases were excluded, mutation calling was performed using following criteria:

1. Both the tumor and normal depths ≥ 100
2. Fisher’s exact test P values < 0.01

Results of the targeted deep sequencing are provided in S3 Table.

### CN profiling

DNA was processed and hybridized to the HumanOmniExpress BeadChip Kit (Illumina). Illumina's GenomeStudio software was used to obtain B allele frequencies (BAF) and log R ratios (LRR) from the raw output data. BAF and LRR were input into the ASCAT algorithm [32] to estimate purity and allele-specific absolute CN, which are used for calculation of CCF. Segmented LRR was also obtained from ASCAT and used for subsequent analyses after the median was shift to 0.

### Identification of founder and progressor CN alterations

To obtain founder and progressor CN alterations, we focused on chromosomal regions subjected to arm-level and focal alterations recurrent among patients, which were reported by the TCGA study [8]. For all the samples in each case, we obtained LRR averaged along each of the chromosomal regions. We assumed that chromosomal regions subjected to founder CN alterations have |LRR| > 0.12 at least in one sample and |LRR| > 0.06 in all the samples. To identify progressor CN alterations, we searched for differentially altered chromosomal regions among every pair of sample groups divided by mutation-based evolutionary tree. The groups were prepared by focusing on branching points in the tree. Note that, except for the first branching point that joins the trunk and branches, each branching point divided samples into two groups: those branching out from the point and the others. For each of the chromosomal regions, we obtained difference of mean LRR between every pair of such groups, and the maximum difference as a statistic, ΔLRR. We then obtained chromosomal regions whose |ΔLRR| > 0.06 as those subjected to progressor CN alterations. For founder and progressor CN alterations identified in this manner, if a chromosomal region subjected to a focal CN alteration is contained by that subjected to an arm-level alteration of the same category, we discarded the former.

### Calculation of cancer cell fraction

Cancer cell fraction (CCF) in which each mutation occurs was estimated by modifying a previously reported approach [33]. Consider a mutation observed in *m* of *n* sequencing reads on a locus of allele-specific absolute CN of *q*_*a*_ and *q*_*b*_ in a sample of purity *α*. The expected allele-fraction *f* of a mutation present in *p* copy in a fraction *c* of cancer cells is calculated by *f (c*, *p*, *α*, *q*_*a*_, *q*_*b*_) = *αcp*(2(1-*α*)+*α*(*q*_*a*_+*q*_*b*_)), with *c*∈[0.01, 1]. Then *P*(*c*|*n*, *m*, *α*, *q*_*a*_, *q*_*b*_) ∝ *Binom*(*m*|*n*, *f*(*c*, *p*, *α*, *q*_*a*_, *q*_*b*_))*P*(*p*|*q*_*a*_, *q*_*b*_)*P*(*c*). For calculating *P*(*p*|*q*_*a*_, *q*_*b*_), we obtained every possible configuration of *p* from *q*_*a*_, and *q*_*b*_, and assumed that they are equally probable. For example, when *q*_*a*_, = *q*_*b*_ = 1, *P*(*p* = 1) = 1. When *q*_*a*_, = 1 and *q*_*b*_ = 2, *P*(*p* = 1) = 2/3 and P(*p* = 2) = 1/3, because a mutation could occur in one copy of allele a, one copy or two copies of allele b. *P*(*c*) is a prior probably of *c* and we assumed a uniform prior. *P*(*c*|*n*, *m*, *α*, *q*_*a*_, *q*_*b*_) was then obtained by calculating these values over a regular grid of 100 *c* values and reported after normalization. We defined the median of *P*(*c*|*n*, *m*, *α*, *q*_*a*_, *q*_*b*_) as CCF.

In this study, *m* ad *n* were obtained from WES data while *α*, *q*_*a*_ and *q*_*b*_ were obtained by ASCAT analysis on SNP array data. For deducing Fig 3D, CCFs were calculated for mutations which occur in sample of which *α* > 0.6. CCF is also used for obtaining clonal and subclonal mutations from single sample sequencing data; we assumed mutations of which CCF exceed 0.8 as clonal mutations, and others as subclonal mutations.

### Mutation analysis using TCGA data

TCGA WES data were processed by the same pipeline used for our WES data. Because only BAM files were available for TCGA data, we first convert BAM files to fastq files. BAM files for COADREAD paired tumor/normal samples were downloaded from https://cghub.ucsc.edu/ with the accession no. 26594–6, which were first converted back to fastq files, where the ‘read1’ and ‘read2’ entries in fastq files were reconstructed based on pair-read information. For reverse strand reads, we generated complemented bases and assigned BaseQuality accordingly. In this process, the reads that had Flag of “not primary alignment”, “read fails platform/vendor quality checks” or “supplementary alignment” were discarded using samtools-0.1.18. Then, the fastq file were input into our WES analysis pipeline to call mutations.

SNP 6.0 array data of COADREAD paired tumor/normal samples were also obtained from https://tcga-data.nci.nih.gov/tcga/. The probe-level signal intensities were converted to BAF and LLR as follows: BAF = *I*_*b*_ /(*I*_*a*_ + *I*_*b*_) and LLR = *I*_*a*_ + *I*_*b*_ where *I*_*a*_ and *I*_*b*_ were signal intensities for A and B alleles. Then, BAF and LLR were input into the ASCAT algorithm to estimate purity and allele-specific absolute CN. In total, 295 COAD and 110 READ TCGA patient samples were available for both the mutation and CN data.

From these data, CCFs were calculated for each mutation to obtain clonal and subclonal mutations. In the analyses of mutational signatures and correlation between patients’ ages and mutation rates, 53 COAD and 5 READ samples whose mutation rates ≥ 10 Mb were separately analyzed, assuming that they belong to the hypermutated subtype.

### Methylation profiling

Genomic DNA was bisulfite treated using the EZ-96 DNA Methylation Kit (Zymo Research Corporation, Orange, CA) and genome-wide DNA methylation were profile with the Illumina HumanMethylation450 BeadChip (Illumina). A methylation level of each probe was then obtained as a β-value. In this study, we focused on only the probes designed for autosomal sequences. In analyses of methylation variance and signature, Positional categories were based on probe information of the HumanMethylation450 BeadChip.

### Identification of founder and progressor methylations

For each probes, differential methylation was quantified with each tumor samples and matched normal tissue by the difference of β-values (Δβ = tumor β value–normal β value). For each case, we assumed that a probe was subjected to founder hyper or hypomethylation, if Δβ > 0.3 or Δβ < 0.3 in all the samples of the case, respectively. Progressor methylations were identified as done for progressor CN alterations; we searched for differentially methylated probes among every pair of the sample groups divided by mutation-based evolutionary tree. For each probe, we obtained difference of mean methylation between every pair of the sample groups, and the maximum difference as a statistic Δ’β. For a threshold value θ, we obtained probes of which |Δ’β| >θ as probes subjected to Progressor methylations. For each case, θ was adjusted so that FDR was 0.1. The FDR was calculated by permutation of the samples. The progressor methylations were further divided into progressor hyper and hypomethylations based on plus and minus of Δβ averaged across the samples. For making heat maps of multiregional methylation profiles in Fig 1, 10000 probes subjected to founder and progressor methylations were randomly sampled, due to the large number of the probes meeting the criteria. Similarly, Fig 4A was made for randomly sampled 6000 probes that had |Δβ| > 0.3 at least one sample of any case, which seems sufficient to provide an overview of the methylomes.

### Analysis of methylation variance

For each probe, we calculated inter- and intra-variance (variance among cases and within cases). Our approach was based on partitioning of the sum of squares, which is utilized in ANOVA. Assuming that each of *k* groups has *n*_*j*_ variables, *x*_*ij*_, the total sum of square *SS*_*T*_ is decomposed into between- and within-group sum of squares, *SS*_*w*_ and *SS*_*b*_:

*SS*_*T*_ = *SS*_*w*_ +*SS*_*b*_, where $ss_{T}=\sum_{j=1}^{k}\sum_{i=1}^{n_{j}}{\left(x_{ij}-\overset{-}{\overset{-}{x}}\right)}^{2}$, $ss_{w}=\sum_{j=1}^{k}\sum_{i=1}^{n_{j}}{\left(x_{ij}-{\overset{-}{x}}_{j}\right)}^{2}$, $ss_{B}=\sum_{j=1}^{k}n_{j}{\left({\overset{-}{x}}_{j}-\overset{-}{\overset{-}{x}}\right)}^{2}$, ${\overset{-}{x}}_{j}=\sum_{i=1}^{n_{j}}x_{ij}/n_{j}$, and $\overset{-}{\overset{-}{x}}=\sum_{j=1}^{k}\sum_{i=1}^{n_{j}}x_{ij}/\sum_{j=1}^{k}n_{j}$.

In our setting, case 2–8 can be regarded as the k groups while the β-values of each sample can be regarded as *x*_*ij*_. Inter- and intra-variance were measured by *SS*_*w*_ and *SS*_*b*_, respectively.

### Expression profiling

mRNA expression profiling were performed with oligonucleotide microarrays (Whole Human Genome Microarray Kit, 4x44K, Agilent Technologies) as described previously [34]. Multiregional gene expression profiles obtained from the microarray experiments were quantile normalized and subjected to the ComBat method [35] to remove batch effects. The EEM method [36] was then applied to the multiregional gene expression profile and the GO and curated Cp entries in MSigDB (http://www.broadinstitute.org/gsea/msigdb/), to obtain module activities of significant expression modules as expression signatures.

### Simulation

#### 1) The BEP model

We computationally simulated BEP employing a cellular automaton model, which assumes each cell in a tumor as a cellular automaton (S16 Fig). A cell has a genome containing *n* genes, each of which is represented as a binary value, 0 (wild) or 1 (mutated). Namely, the genome is represented as a binary vector ***g*** of length *n*. In a unit time step, each cell in the simulated tumor dies with a probability *q*. If the cell does not die, the cell then divides with a probability *p*. Before the cell division, we mutate the genome vector *g*: each of 0 elements of *g* is set to 1 with a probability *r*. The first *d* genes in ***g*** are assumed as driver genes, whose mutations accelerate the division speed. A normal cell without any mutations has a division probability *p*_0_, and acquisition of one driver mutation increases *p* by 10^*f*^-fold in the next time step; i.e., *p* = *p*_0_ · 10^*fk*^, where $k=\sum_{}^{d}g_{i}$, the number of mutated driver genes. The death probability is fixed as *q* = *q*_0_. Let *c* and *t* denote the size of the simulated cell population and the number of the time steps, respectively. We started a simulation with *c*_0_ normal cells and repeated the unit time step while population size *c* ≤ *c*_max_ and time step t ≤ t_max_. A flowchart of the simulation was shown as S21A Fig. In this study, we set parameter values as follows: *n* = 300, *p*_0_ = 0.001, *q*_0_ = 10^−7^, *c*_0_ = 10, *c*_max_ = 10^6^ and *t*_max_ = 5×10^6^. r, *f*, and *d* were subjected to parameter fitting as described below.

#### 2) Simulated tumor growth in a two-dimensional space

We designed a simulated tumor to grow in a two-dimensional square lattice where each cell occupies one lattice point. In the beginning, *c*_0_ cells are initialized as close as possible to the center of the lattice. When a cell dies, the occupied point is cleared and becomes empty. When a cell divides, we place the daughter cell in the neighborhood of the parent cell, assuming the Moore neighborhood (i.e., eight points surrounding a central point). If empty neighbor points exist, we randomly select one point from them. Otherwise, we create an empty point in any of the eight neighboring points by the following procedure. First, for each of the eight directions, we count the number of the consecutive occupied points that range from each neighboring points to immediately before the nearest empty cell as indicated in S21B Fig. Next, any of the eight directions is selected with a probability proportional with 1/*l*_*i*_, where *l*_*i*_ (1 ≤ *i* ≤ 8) is the count of the consecutive occupied points for each direction. The consecutive occupied points in the selected direction are then shifted by one point so that an empty neighboring point appears as shown in S21C Fig. Note that simulation results are dependent on the order of the division operation in the two-dimensional square lattice. We first marked cells to be divided and applied the division operation to the marked cells along an outward spiral starting from the center. In each round on the spiral, the direction was randomly flipped in order to keep spatial symmetry. An example of such spirals was shown in S21D Fig.

#### 3) In silico multiregional sequencing

We also performed multiregional sequencing of the simulated tumor in silico. We assumed 25 small square lattices of 31×31 that are evenly distributed in the whole square lattice where the tumor was spread (e.g. boxes in S21E Fig). To obtain multiregional profiles of *s* samples, *s* small square lattices were randomly selected from the small square lattices where at least a half of lattice points were occupied by cells (e.g. filled boxes in S21E Fig). In each of the *s* regions, the proportions of mutated cells for each gene were obtained as a VAF. We then set VAFs that do not exceed 0.3 were set to 0; this filtering step was based on an assumption that multiregional sequencing misses low-frequency variants. Finally, the VAFs were represented as an *n*×*s* multiregional mutation profile matrix whose rows and columns represent genes and samples, respectively.

#### 4) Parameter fitting

We fitted parameters of the BEP model to the real data by employing an approximate Bayesian computation approach [37]. Since the real multiregional mutation profiles were commonly characterized by the presence of founder and unique mutations, we focused on the proportion of founder and unique mutations per sample as summary statistics, φ and θ. Namely, for a given multiregional mutation profile matrix, φ is obtained as the number of rows (genes) which have non-zero elements in all columns (samples), while θ is obtained by counting the number of rows which have non-zero elements uniquely in each column and averaging the number over the 5 columns.

We first obtained observed values of the summary statistics in the real multiregional mutation profiles of our 9 cases. Since φ and θ are dependent on the number of multiregional samples, we fixed *s* to 5, which is the minimum number of samples in the 9 cases. For case 4 and 9, which contained 5 samples in each, we simply calculated φ and θ. For the other samples that contained more than 5 samples, we performed downsampling to obtain 10 mutation profile matrices of 5 samples, and averaged φ and θ over the 10 trials. S17A Fig shows φ and θ while S17B Fig shows multiregional mutation profiles for each case. From φ and θ of the 9 cases, we estimated the observed values as φ = 0.718±0.115 and θ = 0.138±0.040 (mean±standard deviation).

We next performed the simulation with different parameter settings to evaluate which parameter setting leads to summary statistic values similar to the observed ones. Here, d (the number of driver genes), f (strength of driver genes) and r (mutation rate) were subjected to parameter fitting analysis. We prepared 10 integers from 1 to 10 as *d*; 10 numbers from 0.1 to 1.0 incremented by 0.1 as *f*; and 0.0001, 0.0003, 0.001, 0.003, and 0.01 as *r*, and take every combination of the parameter values as done in grid search. This leads to 10 × 10 × 5 = 500 parameter settings, for each of which the BEP simulation was repeated 50 times. For the 50 simulated tumors from each parameter setting, we then performed in silico multiregional sequencing with *s* = 5 to obtain φ and θ. Finally, for each parameter setting, the proportion of instances whose statistics (both φ and θ) fall within 1 standard deviation from the mean of the observed values was calculated and visualized as heat maps in S18A Fig. From this data, we found that the BEP model can produce multiregional profiles similar to those of our 9 cases if a high mutation rate, a sufficient number and sufficient strength of driver genes (e.g., r = 0.01, d≧0.4 and f≧0.8) are assumed. S18B Fig shows representative multiregional mutation profile matrices from simulations with such parameter settings.

#### 5) Analysis and visualization of simulation data

Based on the parameter fitting analysis, we fixed parameter as r = 0.01, d = 6, and f = 0.8 in the following analysis. From a tumor simulated with this parameter setting, we randomly sampled *m* cells (here, we set m = 500) to obtain an *n*×*m* single-cell mutation profile matrix, each of whose *m* columns is the *n*-dimensional genome vector ***g*** for each cell. We obtained color labels for each cell, as done for the sample color labels in the real data. Namely, we applied principal component analysis to the single-cell mutation profile matrix and mixed RGB colors according to dimension-reduced genome vectors. Each cell on the two-dimensional square lattice was colored with the corresponding color label (Fig 5A). The single-cell mutation profile matrix was visualized as a cluster heat map together with the color labels on the top (Fig 5B). We also applied in silico multiregional profiling with s = 8 to obtain a multiregional mutation profile matrix, which was also visualized as a heat map (Fig 5C). Its color labels were prepared using the PCA loading vectors calculated from the single-cell mutation profile matrix. Evolutionary snapshots of the same tumor were presented in S20 Fig while four other instances from independent simulation trials were presented in S19 Fig. To calculate the distribution of VAFs (Fig 5D) and proportion of driver genes (Fig 5E) in different categories of mutations, 20 multiregional mutation profile matrices were obtained from independent simulation trials. Rows of the 10 matrices were then categorized into “founder” (having non-zero elements in all columns), “shared” (having non-zero elements in not all but multiple columns), “unique” (having non-zero elements uniquely in any column), and “unmutated” (having zero elements in all columns). The distribution of VAFs was obtained by extracting non-zero elements from the rows of each category. The proportion of driver genes was obtained by counting rows associated with driver genes in the rows of each category.

## Supporting Information

S1 Fig Depth of whole exome sequencing. (A) Mean depth of each sample. (B) Fractions of the target region covered with at least 10x, 20x and 30x depth.
(PDF)

S2 Fig Evidence of subclonal mixing. We searched for progressor mutations that were shared by distant branches in the evolutionary trees of the 9 cases, and found clear examples of such singular mutations in case3 and case7. The heat maps show VAFs of the singular mutations from exome and targeted deep sequencing for case3 and case7, respectively. Timings of subclonal mixing events were inferred and indicated by red arrows on the evolutional trees.
(PDF)

S3 Fig Pathway-level view of genomic ITH. (A) The color table shows whether any member of the 5 driver pathways was disrupted by founder and progressor genomic alterations (i.e., mutations or focal CN alterations) in the 9 cases. The driver pathways and their members were obtained from the TCGA paper [8]. (B) Genomic alteration profiles of the driver pathway members in each of the 9 cases.
(PDF)

S4 Fig The number of founder, shared and unique mutations. (PDF)

S5 Fig Correlations between mutation counts and patients’ ages. In addition to our data, multiregional mutation data from previous clear cell renal cell carcinoma [3] and pancreatic cancer [2] studies were analyzed. ρ’s are Spearman’s correlation coefficients.
(PDF)

S6 Fig Validation for using clonal and subclonal mutations as surrogates of founder and progressor mutations. (**A**) The Sciclone analysis [38] was performed on three representative samples. (**B**) For each of the clonal and subclonal mutations estimated based on cancer cell fraction, proportions of founder, shared and unique mutations were presented. This analysis was performed for samples whose purities exceed 0.6. The result demonstrated that, when we focused on each single sample, founder and progressor mutations tended to exist as clonal and subclonal mutations, respectively.
(PDF)

S7 Fig Mutational analysis using TCGA data. (**A**) Correlations between mutation rates and patients’ ages. P-values were calculated by The Wilcoxon rank-sum test. (**B**) Mutational signature analysis. (**C**) For each base substitution type, the number of patients having the larger number of clonal or subclonal mutations was shown. P-values were calculated by the binomial test.
(PDF)

S8 Fig Mutational signatures in the trinucleotide context. (A) Founder and progresor mutations in the 9 cases were divided into 96 substitution patterns based on mutated bases and their 5’ and 3’ flanking bases, and the percentages of each substitution pattern were plotted as a bar plot. (B) Clonal and subclonal mutations in the TCGA samples were analyzed as in A.
(PDF)

S9 Fig Multiregional CN profiles. The heat maps show LRR across chromosomes in each sample.
(PDF)

S10 Fig Density plot showing distributions of inter- and intratumor variance for each category of probes. (PDF)

S11 Fig Classification of the CIMP and non-CIMP subtypes. Methylation datasets of 529 TCGA COADREAD samples were obtained from https://tcga-data.nci.nih.gov/tcga/. For the 2000 probes showing the highest variance in the TCGA samples, we made clustered heat maps of β values for only the TCGA samples (**A**) and the TCGA samples mixed with our samples (**B**). Based on this result, we classified cases 5 and 9 into the CIMP subtype.
(PDF)

S12 Fig Differential contribution of different types of epigenetic domains to intratumor and intertumor variance. Enrichment scores for intratumor (A) and intertumor (B) variance were calculated as in Fig 4B. Classification of chromosomal regions was based epigenetic status in normal colon tissue, which is profiled by the NIH Roadmap Epigenomics Consortium [13].
(PDF)

S13 Fig The number of probes subjected to founder and progressor methylation. (PDF)

S14 Fig Multiregional methylation profiles of epigenetic marker genes. The heat map shows Δβ values of probes contained by the CpG island promoters of the epigenetic gatekeepers [14] and CIMP-related genes [39].
(PDF)

S15 Fig Correlations between methylation and patients’ ages in the TGGA samples. We assumed that hyper- and hypomethylated probes have Δβ> 0.3 and Δβ< -0.3, respectively. CpG island hypermethylation was significantly correlated with patients’ ages. r’s are Pearson’s correlation coefficients and p-values were calculated by the Pearson's correlation test.
(PDF)

S16 Fig A schema of the BEP model. A cell has n genes, d out of which are driver genes. In this schema, n = 10 and d = 4, and red and blue boxes denote driver and non-drive genes, respectively. In a unit time step, a cell divides or dies with probabilities p and q, respectively. During each cell division, each gene is randomly mutated with a probability r, and one driver mutation, which is denoted by a red cross, increases p by 10^f^ -fold.
(PDF)

S17 Fig Parameter fitting 1. (**A**) Observed values of summary statistics in the real data. The proportion of founder and unique mutations were obtained for 9 cases. For each of the cases except case4 and case9, downsamplings were performed to obtain 10 multiregional profiles of 5 samples and the statistics were averaged over the downsampling trials. The error bars indicate standard deviations for the downsampling trials. Finally, an “average” over the 9 cases was obtained as an estimate of the observed value of each summary statistic. The error bars at “average” indicate standard deviations over the 9 cases. (**B**) Multiregional mutation profiles from the real experiments. For the cases except case4 and case9, representative samples from the 10 downsampling trials were presented as in Fig 5C.
(PDF)

S18 Fig Parameter fitting 2. (**A**) The proportion of simulation instances fitted to the real data. The proportion of simulation instances whose statistics fall within 1 standard deviation from the mean of the observed values was calculated for each parameter settings and visualized as heat maps (**B**) Multiregional mutation profiles from the simulations. Representative instances from simulation with indicated parameter settings were presented as in Fig 5C. Left blue bars indicate driver genes.
(PDF)

S19 Fig Four tumors from independent simulation trials. Simulated tumors, simulated single-cell and multiregional mutation profile matrix from four simulation trials are shown as in Fig 5A–5C.
(PDF)

S20 Fig Simulated tumor growth. (**A**) Snap shots of growing tumors in a simulation. Differently colored cell populations represent each clone. (**B**) A growth curve of the simulated tumor. The snap shots were obtained at each plotted point. (**C**) Single-cell mutation profiles of the simulated tumor during growth. Timing at which the mutation profiles were obtained are indicated by red rectangles in (**A**) and red plotted points in (**B**). Top colored bars represent each clone while left blue bars represent driver genes.
(PDF)

S21 Fig Illustration of our simulation method. (**A**) A flowchart of our simulation. (**B**, **C**, **D**, and **E**,) illustration of division operation. See the simulation section in Materials and Methods.
(PDF)

S1 Table Information of 9 colorectal cancer cases. (XLSX)

S2 Table Information of 84 analyzed samples. (XLSX)

S3 Table Information of detected mutations. (XLSX)
