# Correction: Integrated Multiregional Analysis Proposing a New Model of Colorectal Cancer Evolution

**DOI:** 10.1371/journal.pgen.1006798

**Published:** 2017-05-19

**Authors:** Ryutaro Uchi, Yusuke Takahashi, Atsushi Niida, Teppei Shimamura, Hidenari Hirata, Keishi Sugimachi, Genta Sawada, Takeshi Iwaya, Junji Kurashige, Yoshiaki Shinden, Tomohiro Iguchi, Hidetoshi Eguchi, Kenichi Chiba, Yuichi Shiraishi, Genta Nagae, Kenichi Yoshida, Yasunobu Nagata, Hiroshi Haeno, Hirofumi Yamamoto, Hideshi Ishii, Yuichiro Doki, Hisae Iinuma, Shin Sasaki, Satoshi Nagayama, Kazutaka Yamada, Shinichi Yachida, Mamoru Kato, Tatsuhiro Shibata, Eiji Oki, Hiroshi Saeki, Ken Shirabe, Yoshinao Oda, Yoshihiko Maehara, Shizuo Komune, Masaki Mori, Yutaka Suzuki, Ken Yamamoto, Hiroyuki Aburatani, Seishi Ogawa, Satoru Miyano, Koshi Mimori

The Data Availability Statement for this article is incorrect. The correct statement is: Sequence data are available from the DNA Data Bank of Japan (DDBJ) under the accession number JGAS00000000060 (https://ddbj.nig.ac.jp/jga/viewer/view/study/JGAS00000000060) for researchers who meet the criteria for access to confidential data. Array data have been deposited to the Gene Expression Omnibus database, with the accession number GSE90709 (https://www.ncbi.nlm.nih.gov/geo/query/acc.cgi?acc=GSE90709).

There is an error in the third sentence of the Abstract. The phrase ‘intertumor heterogeneity’ is incorrect, and should read ‘intratumor heterogeneity’. The correct sentence is: ‘Extensive intratumor heterogeneity is observed, from which we inferred the evolutionary history of the tumors.’
