# Supplementary figures and images for: Integrated Multiregional Analysis Proposing a New Model of Colorectal Cancer Evolution
Source: PLoS Genet. 2016 Feb 18;12(2):e1005778. doi: 10.1371/journal.pgen.1005778 (PMC4758664; doi:10.1371/journal.pgen.1005778)

**A**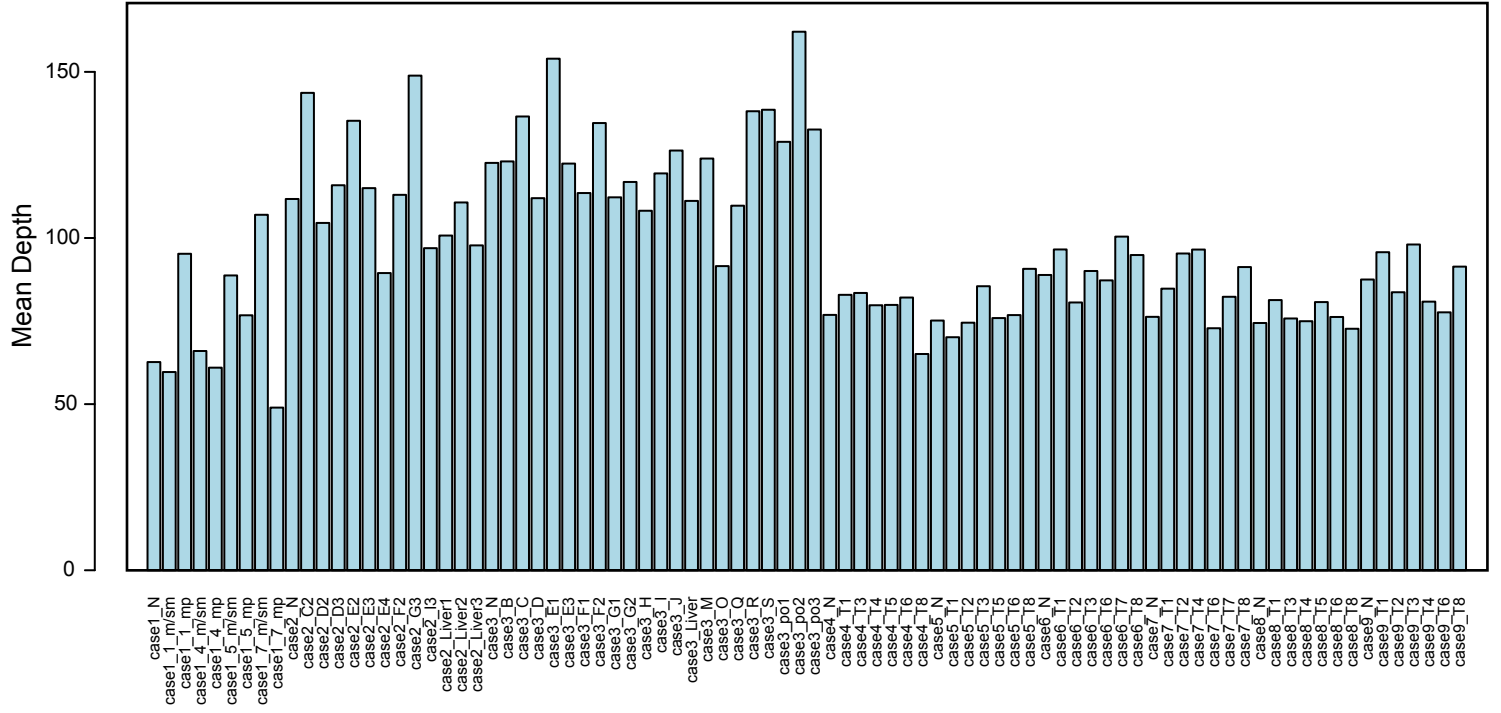**B**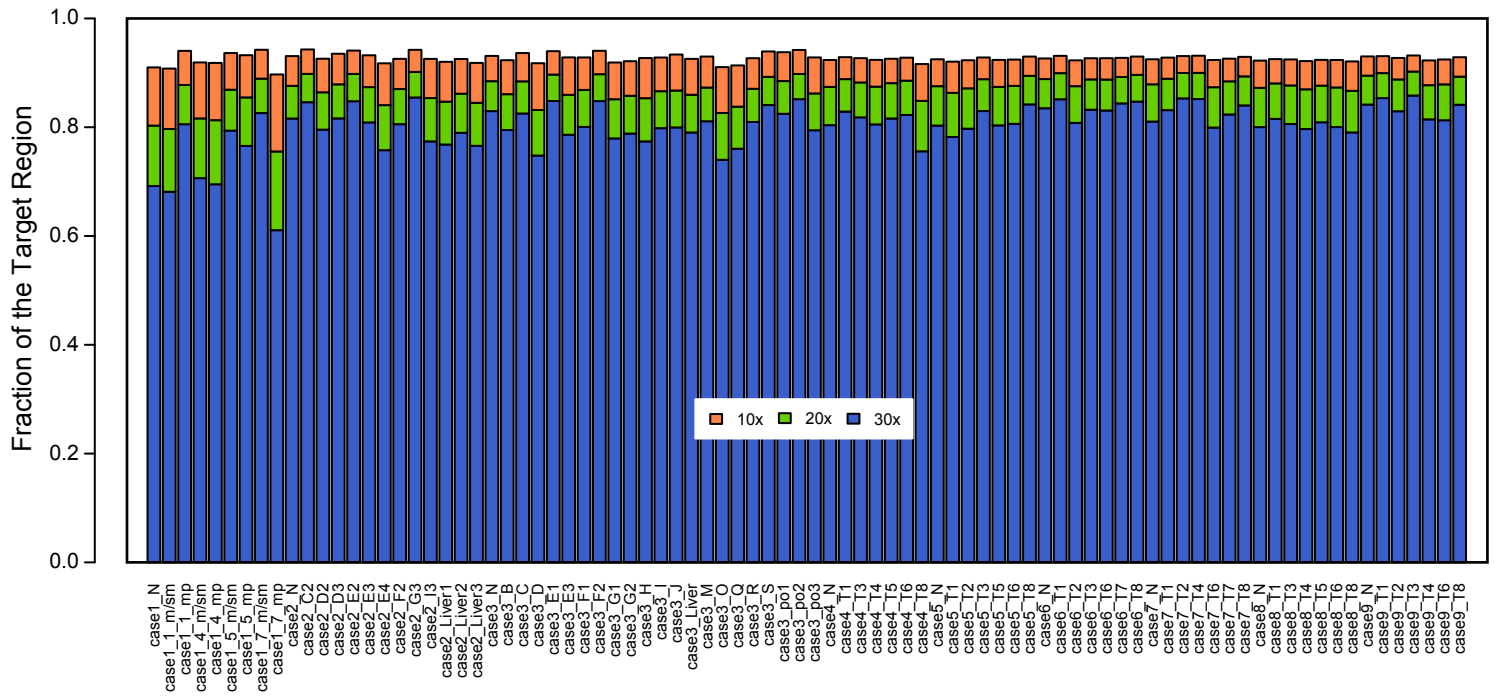

Supplement: S1 Fig — (A) Mean depth of each sample. (B) Fractions of the target region covered with at least 10x, 20x and 30x depth. (PDF) [file pgen.1005778.s001.pdf]

## case3

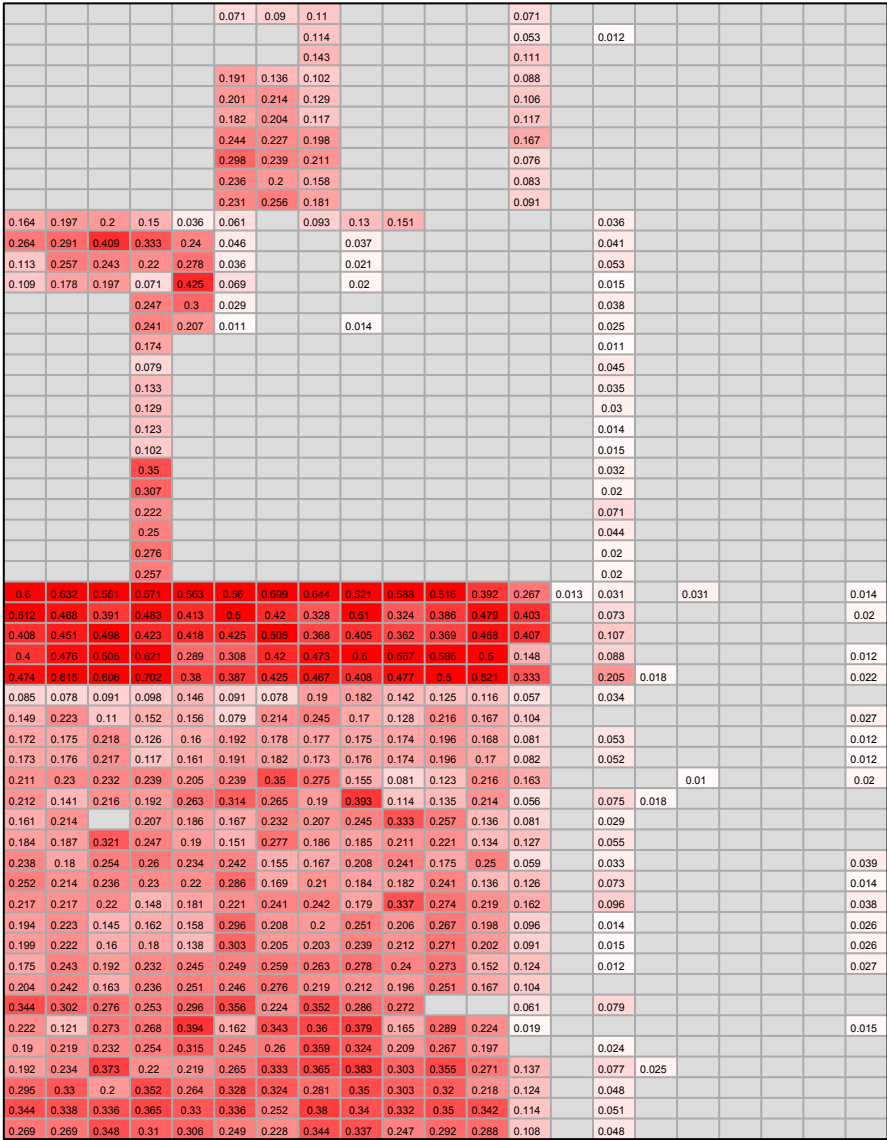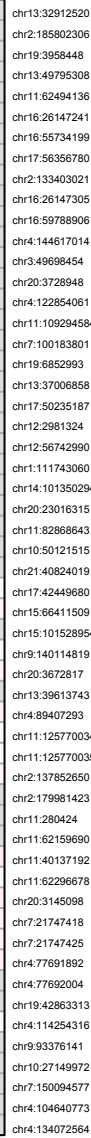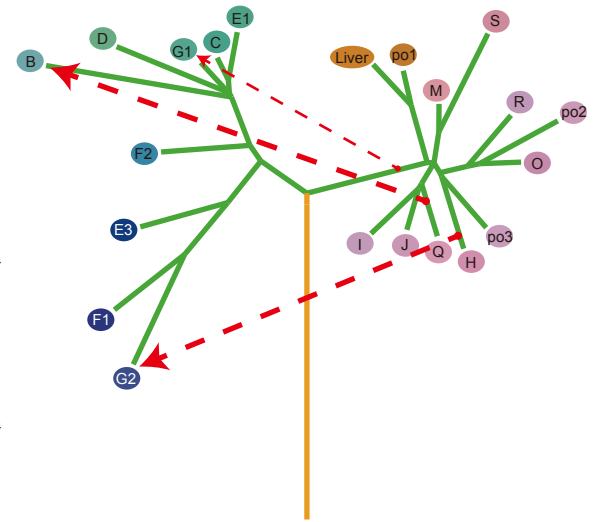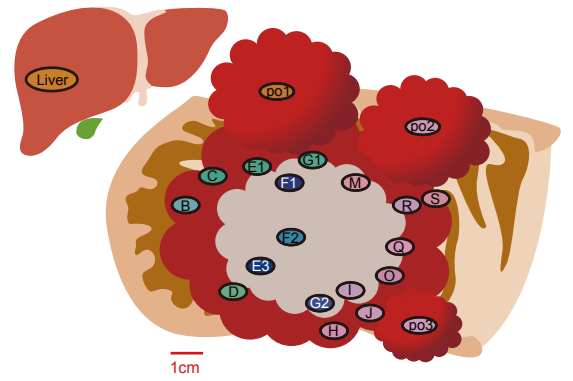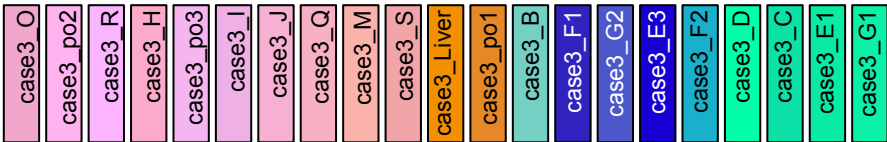

case7

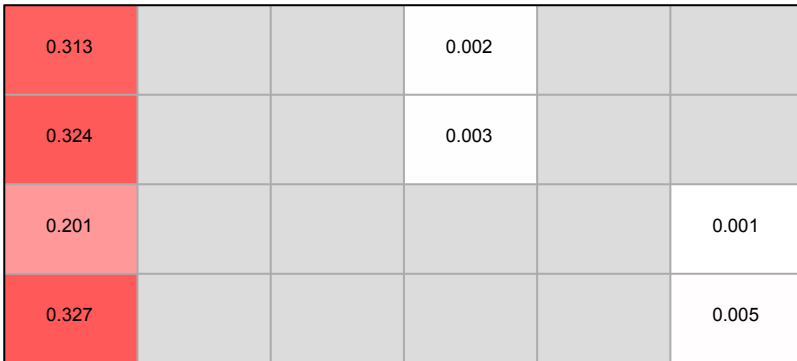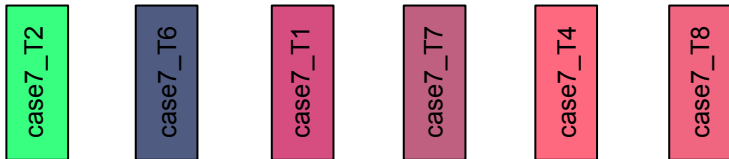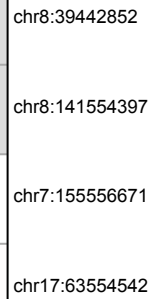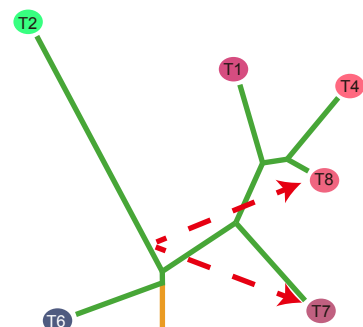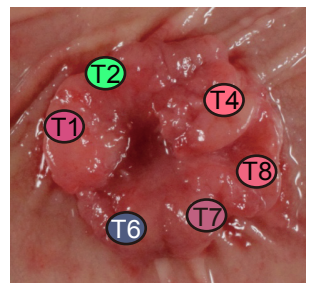

1 cm

Supplement: S2 Fig — We searched for progressor mutations that were shared by distant branches in the evolutionary trees of the 9 cases, and found clear examples of such singular mutations in case3 and case7. The heat maps show VAFs of the singular mutations from exome and targeted deep sequencing for case3 and case7, respectively. Timings of subclonal mixing events were inferred and indicated by red arrows on the evolutional trees. (PDF) [file pgen.1005778.s002.pdf]

**A**

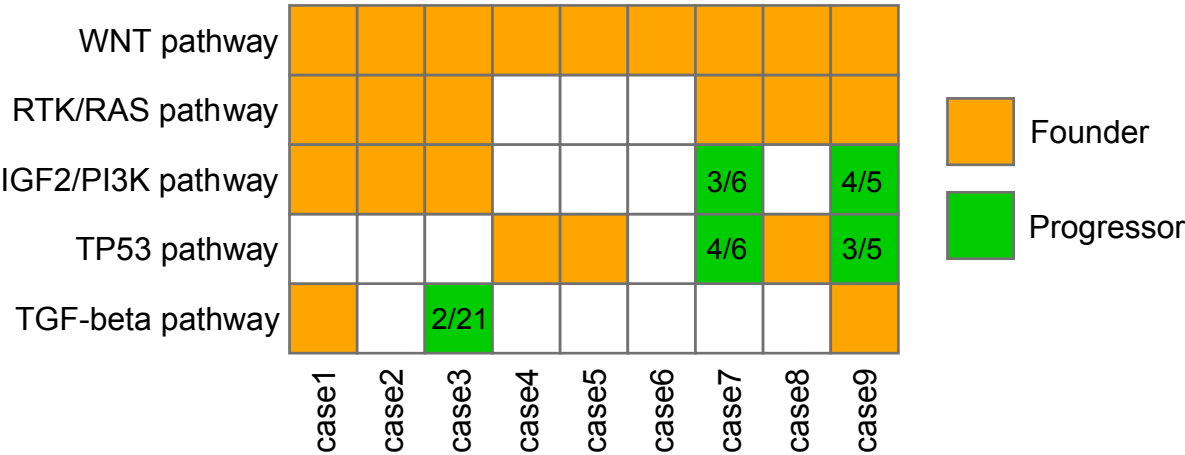

**B**

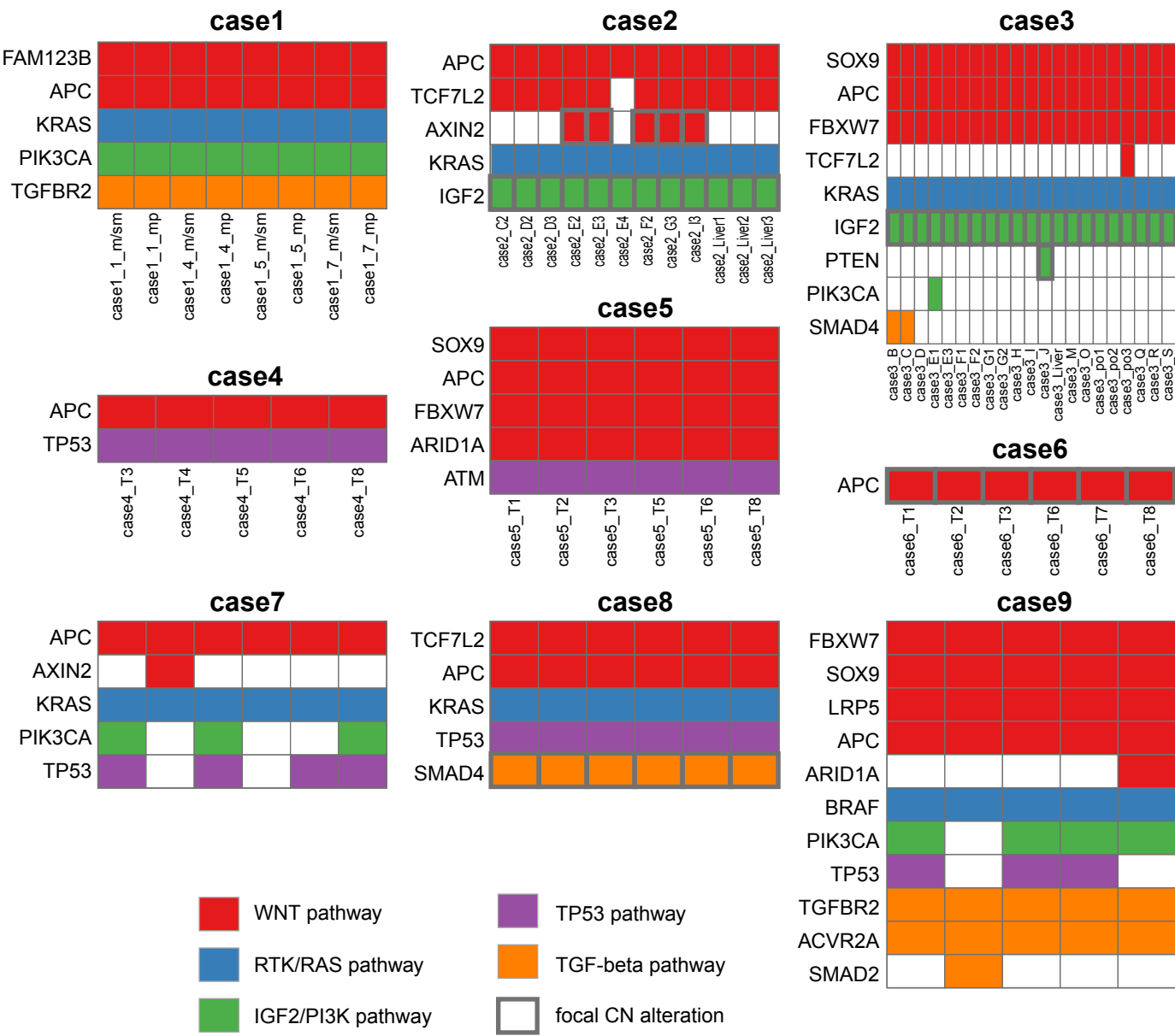

Supplement: S3 Fig — (A) The color table shows whether any member of the 5 driver pathways was disrupted by founder and progressor genomic alterations (i.e., mutations or focal CN alterations) in the 9 cases. The driver pathways and their members were obtained from the TCGA paper [8]. (B) Genomic alteration profiles of the driver pathway members in each of the 9 cases. (PDF) [file pgen.1005778.s003.pdf]

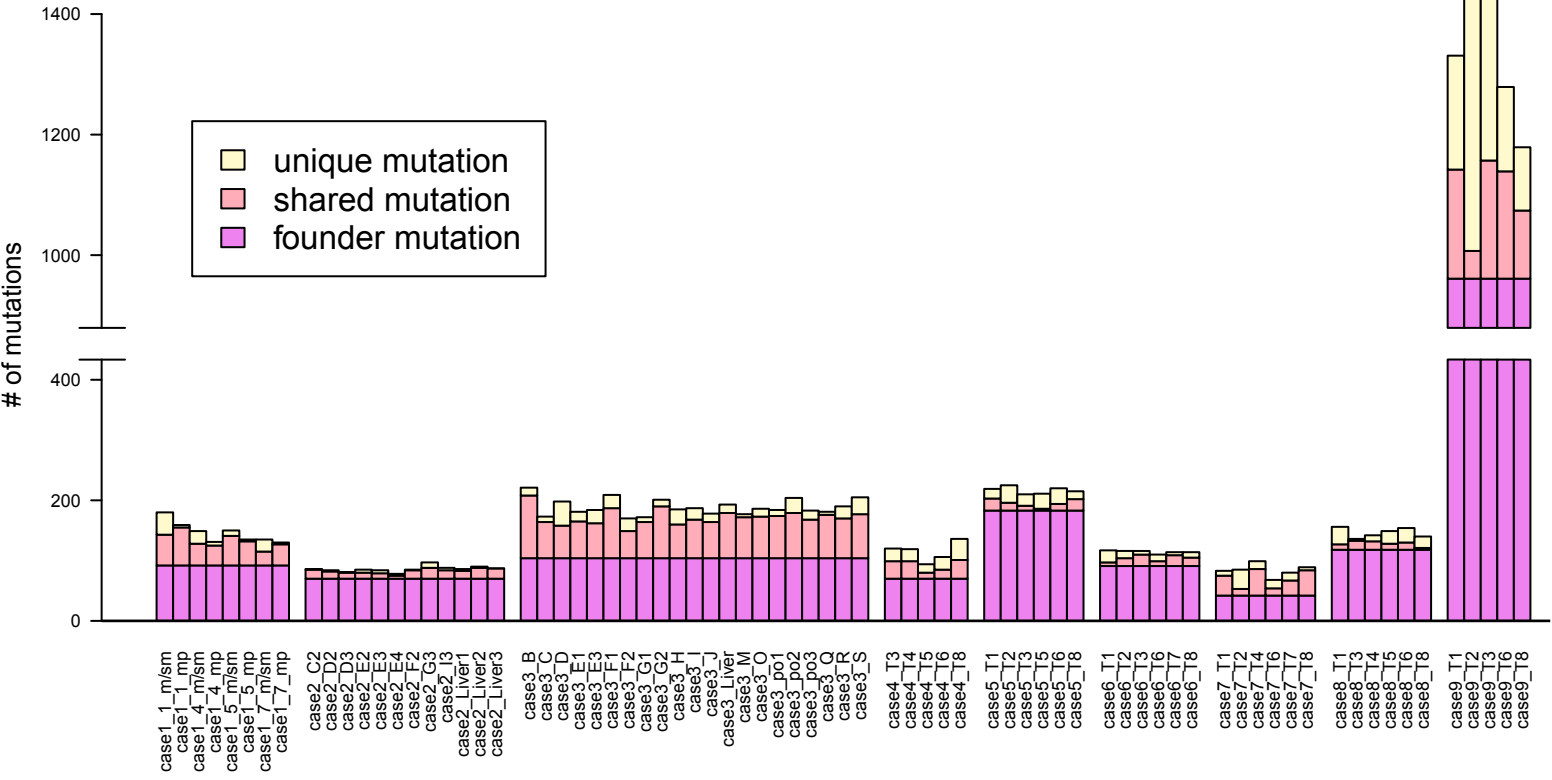

Supplement: S4 Fig — (PDF) [file pgen.1005778.s004.pdf]

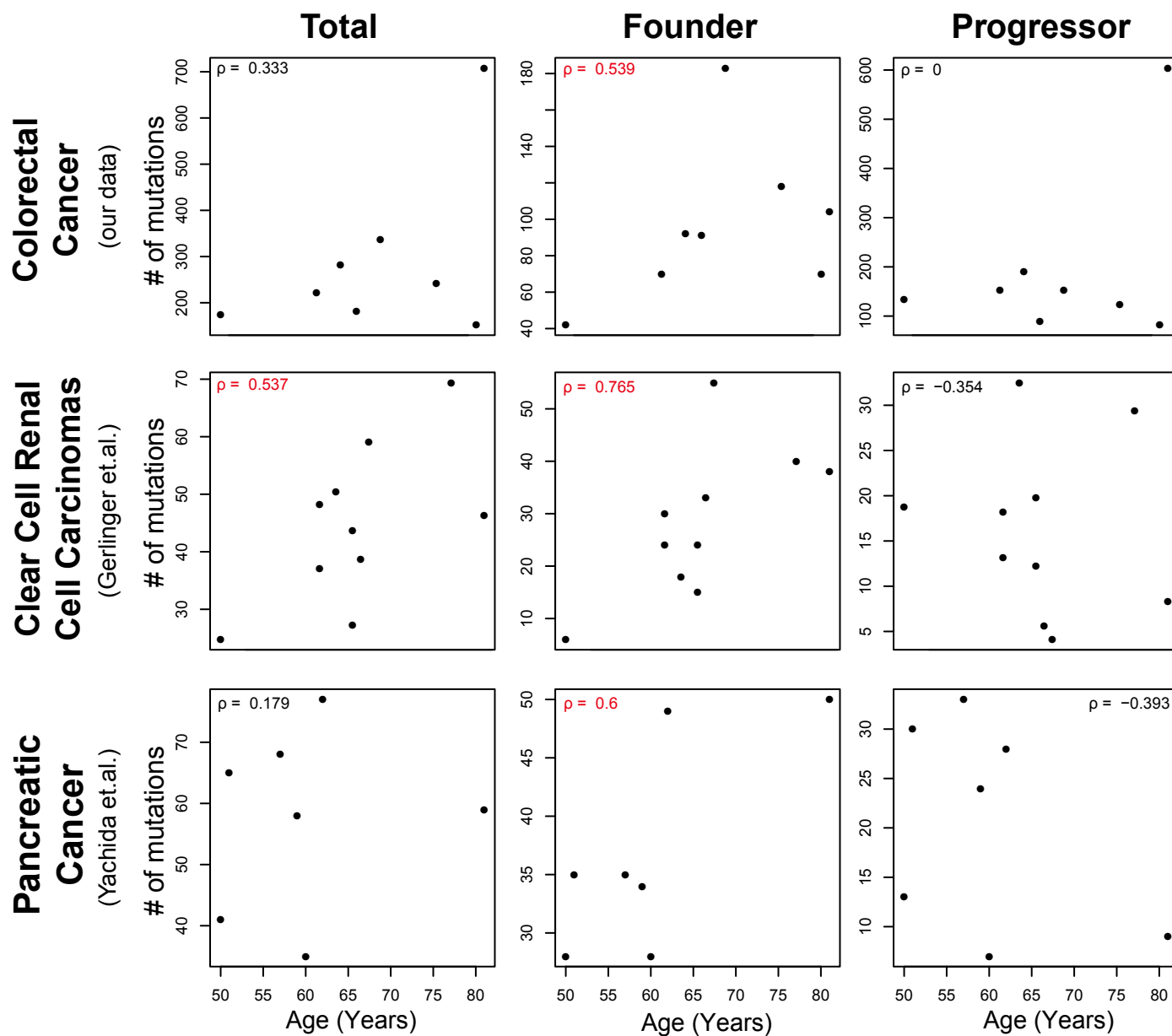

Supplement: S5 Fig — In addition to our data, multiregional mutation data from previous clear cell renal cell carcinoma [3] and pancreatic cancer [2] studies were analyzed. ρ’s are Spearman’s correlation coefficients. (PDF) [file pgen.1005778.s005.pdf]

**A**

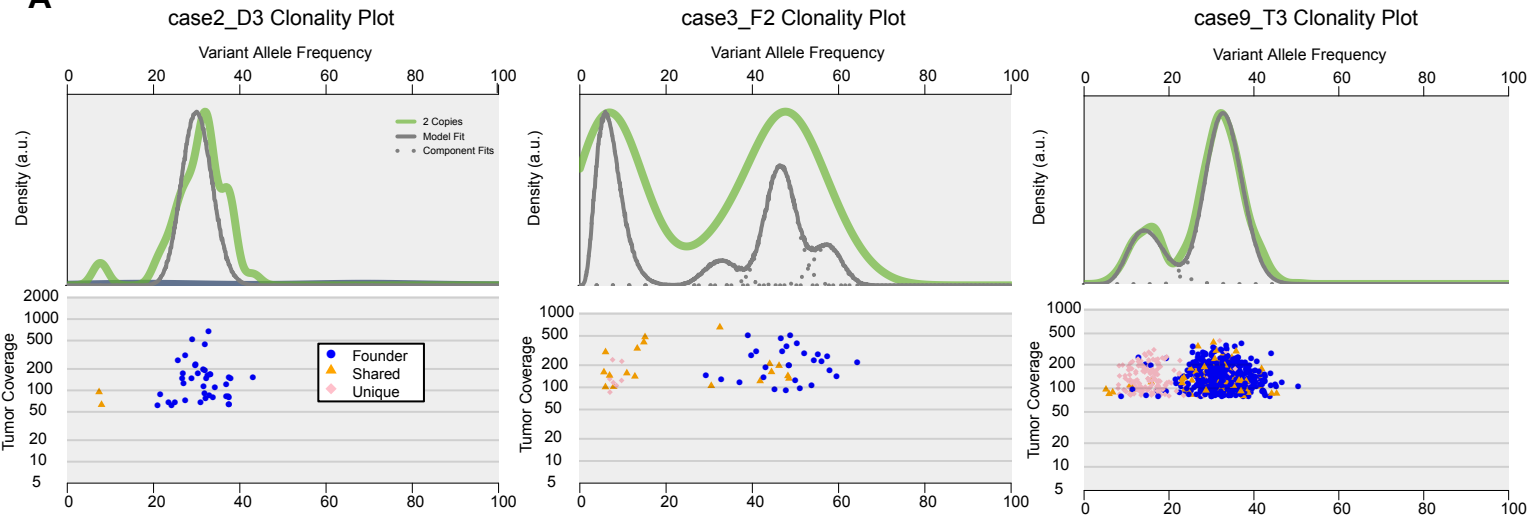

**B**

**Clonal Mutations**

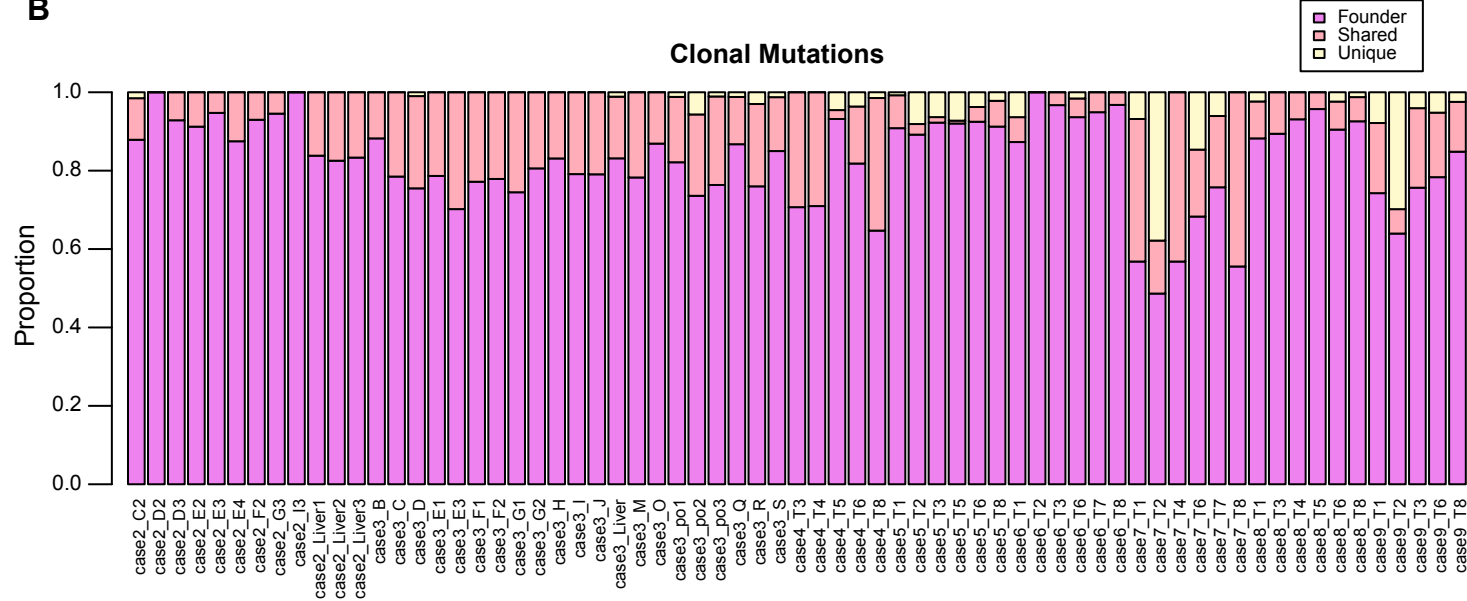

**Sub Clonal Mutations**

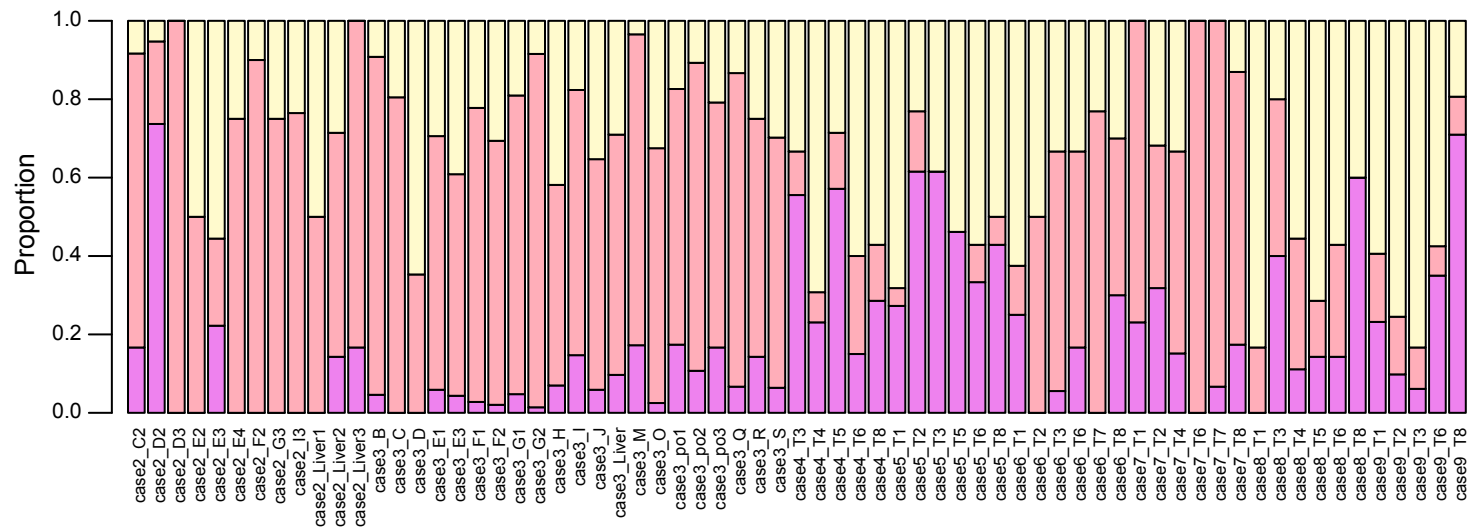

Supplement: S6 Fig — (A) The Sciclone analysis [38] was performed on three representative samples. (B) For each of the clonal and subclonal mutations estimated based on cancer cell fraction, proportions of founder, shared and unique mutations were presented. This analysis was performed for samples whose purities exceed 0.6. The result demonstrated that, when we focused on each single sample, founder and progressor mutations tended to exist as clonal and subclonal mutations, respectively. (PDF) [file pgen.1005778.s006.pdf]

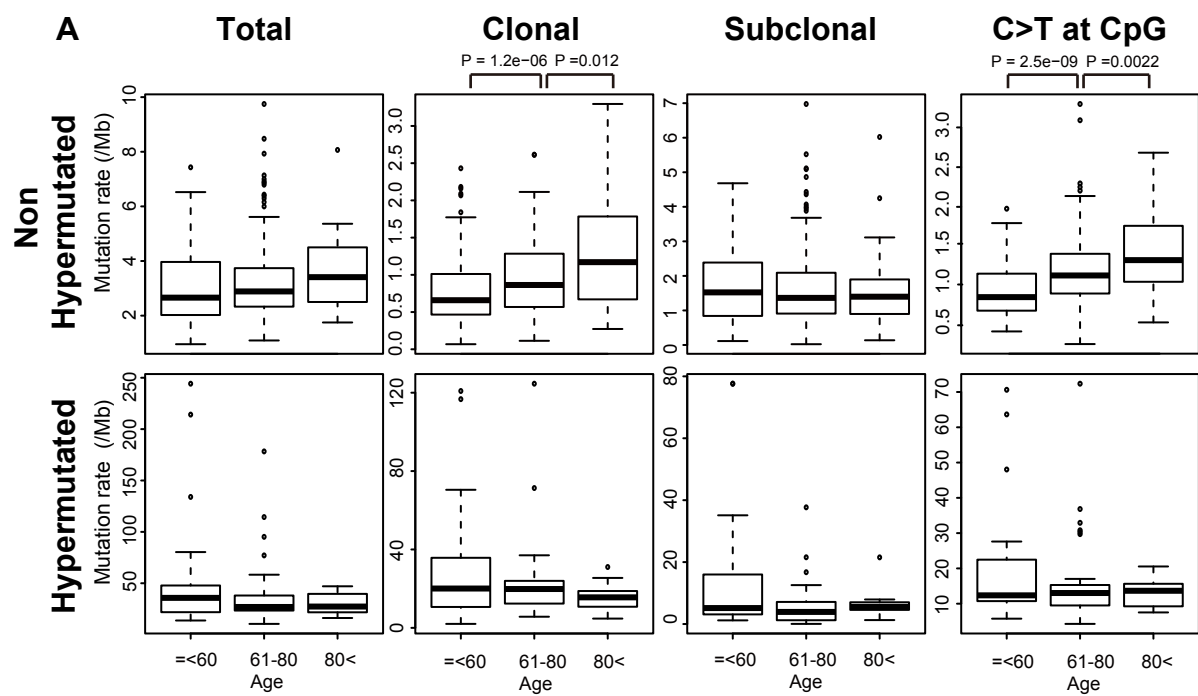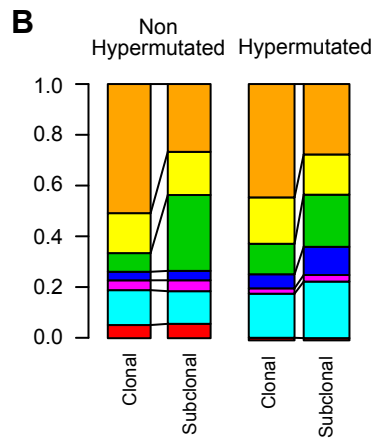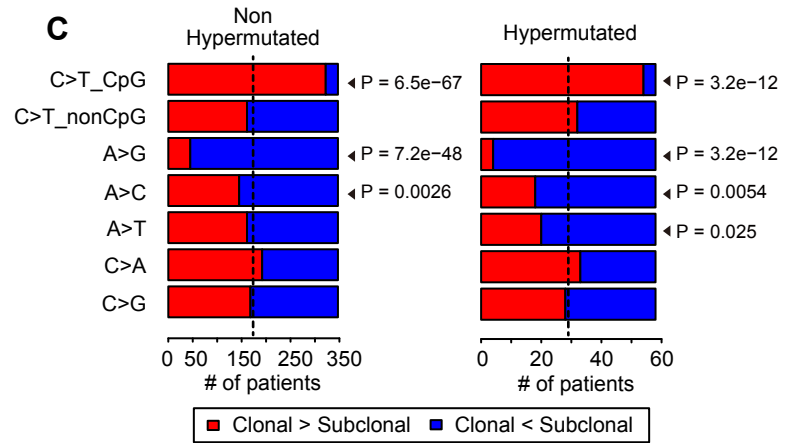

Supplement: S7 Fig — (A) Correlations between mutation rates and patients’ ages. P-values were calculated by The Wilcoxon rank-sum test. (B) Mutational signature analysis. (C) For each base substitution type, the number of patients having the larger number of clonal or subclonal mutations was shown. P-values were calculated by the binomial test. (PDF) [file pgen.1005778.s007.pdf]

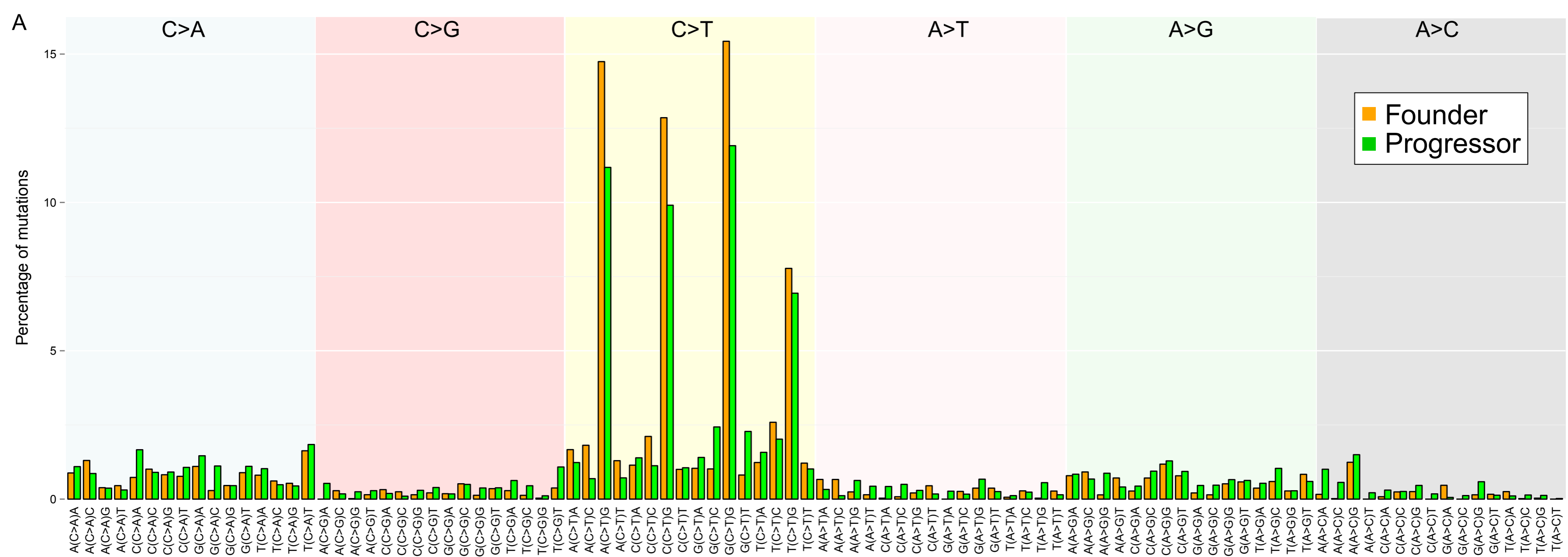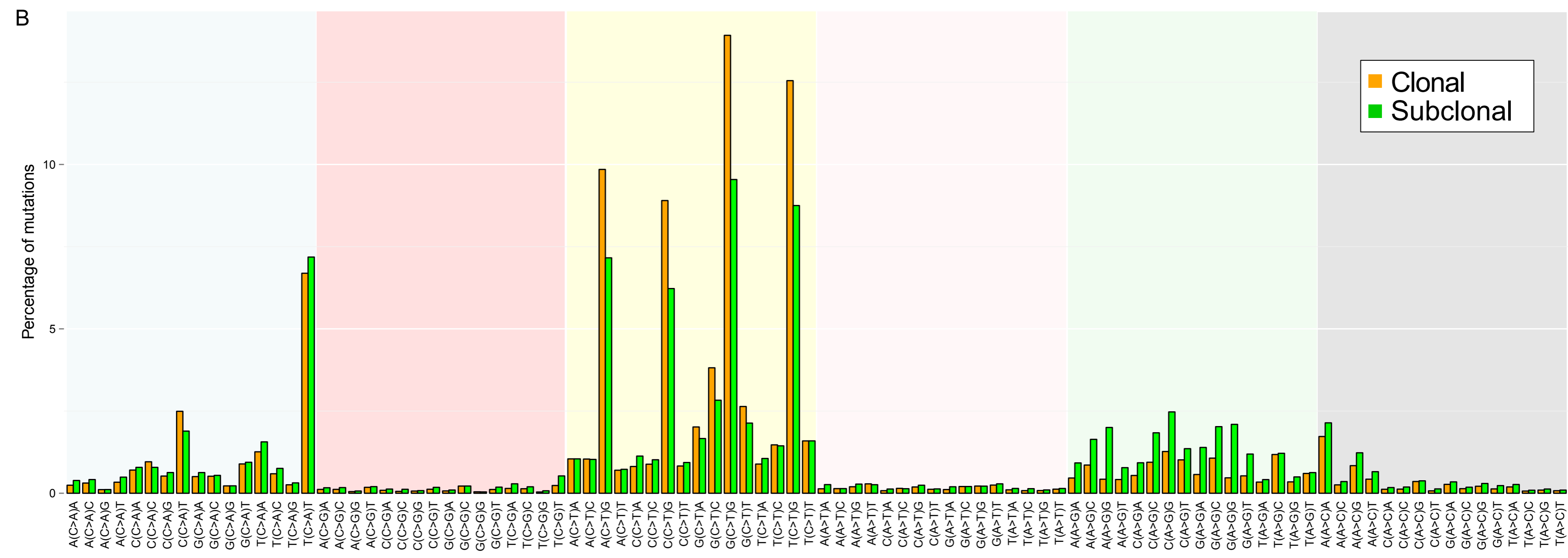

Supplement: S8 Fig — (A) Founder and progresor mutations in the 9 cases were divided into 96 substitution patterns based on mutated bases and their 5’ and 3’ flanking bases, and the percentages of each substitution pattern were plotted as a bar plot. (B) Clonal and subclonal mutations in the TCGA samples were analyzed as in A. (PDF) [file pgen.1005778.s008.pdf]

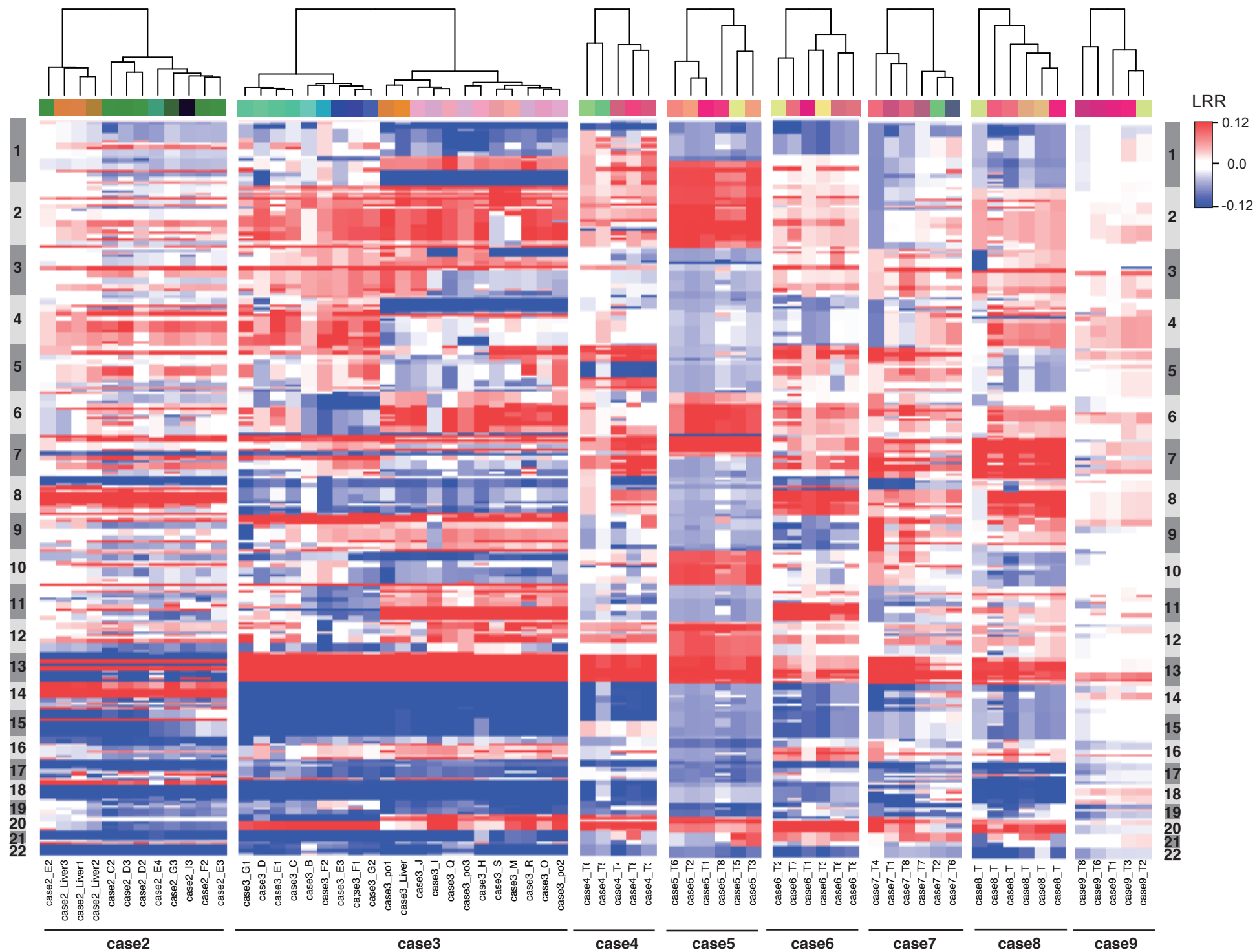

Supplement: S9 Fig — The heat maps show LRR across chromosomes in each sample. (PDF) [file pgen.1005778.s009.pdf]

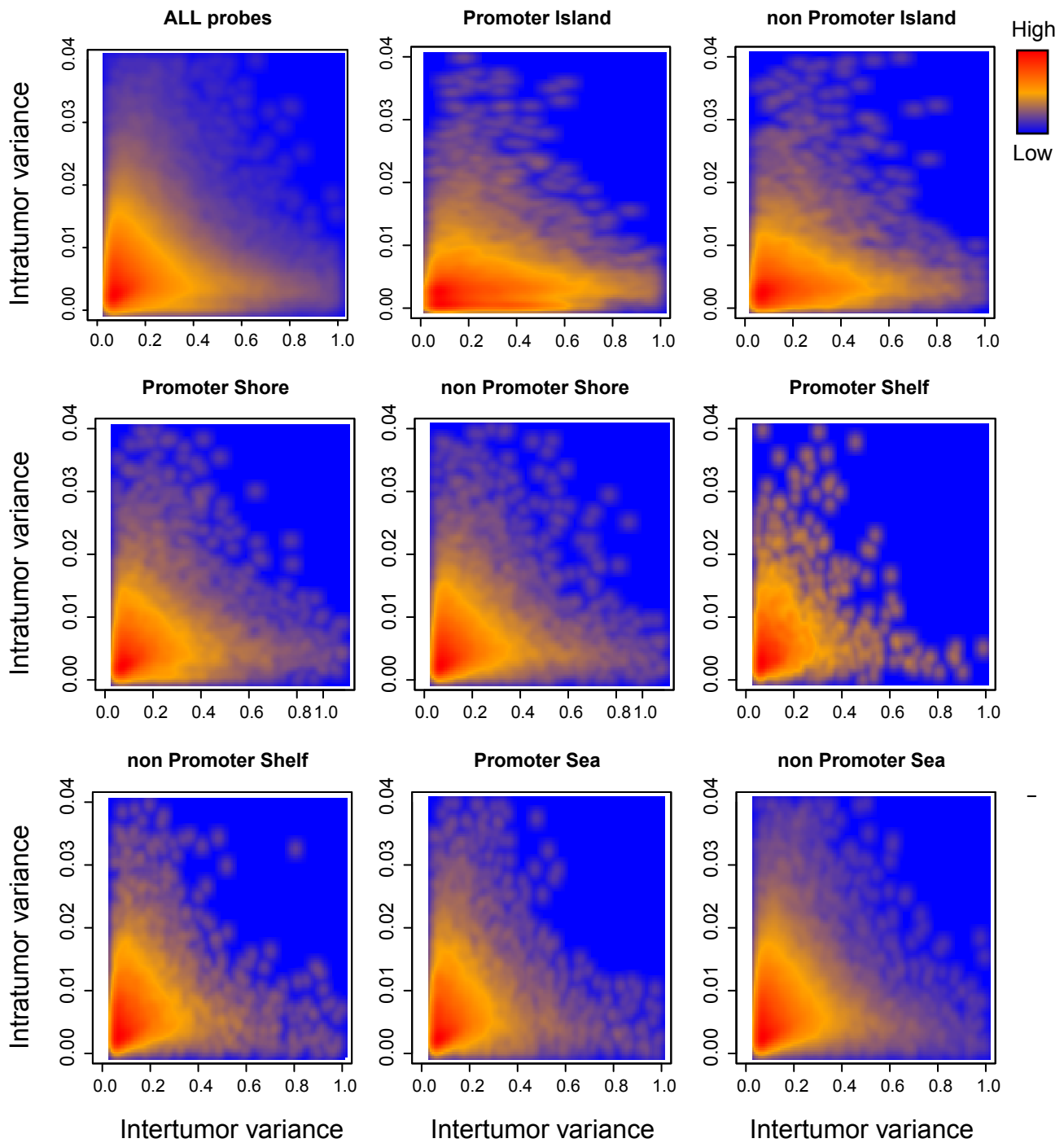

Supplement: S10 Fig — (PDF) [file pgen.1005778.s010.pdf]

**A**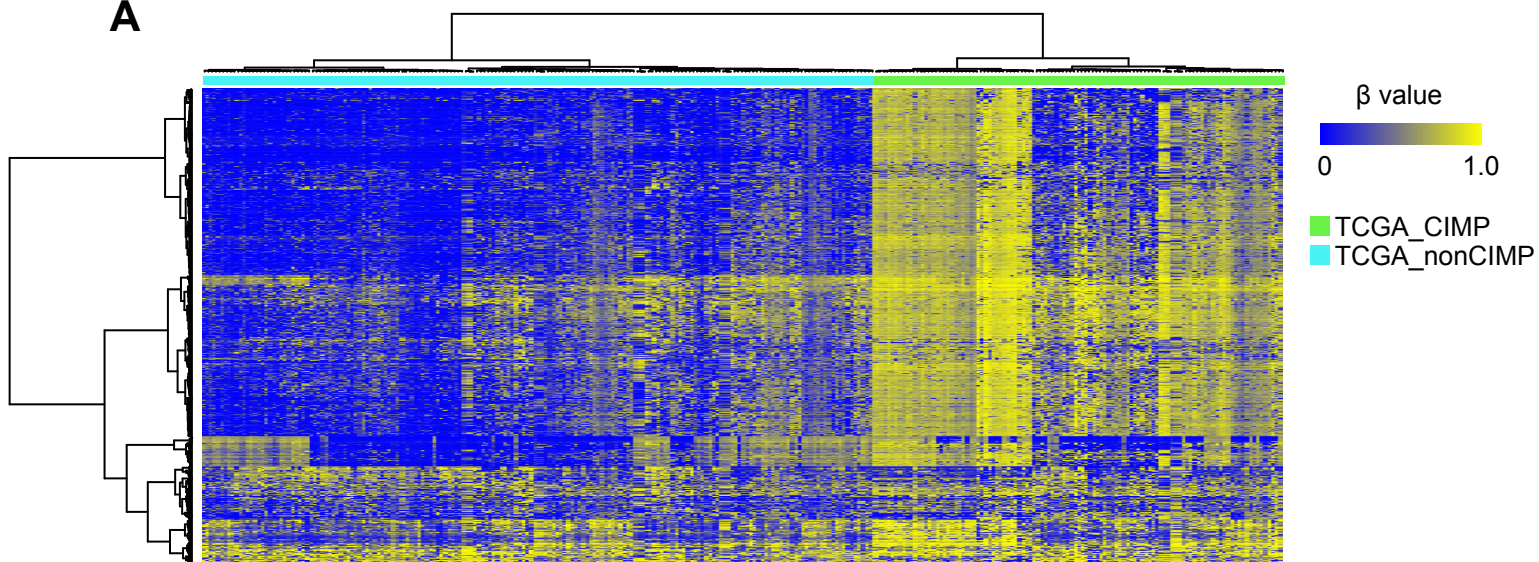**B**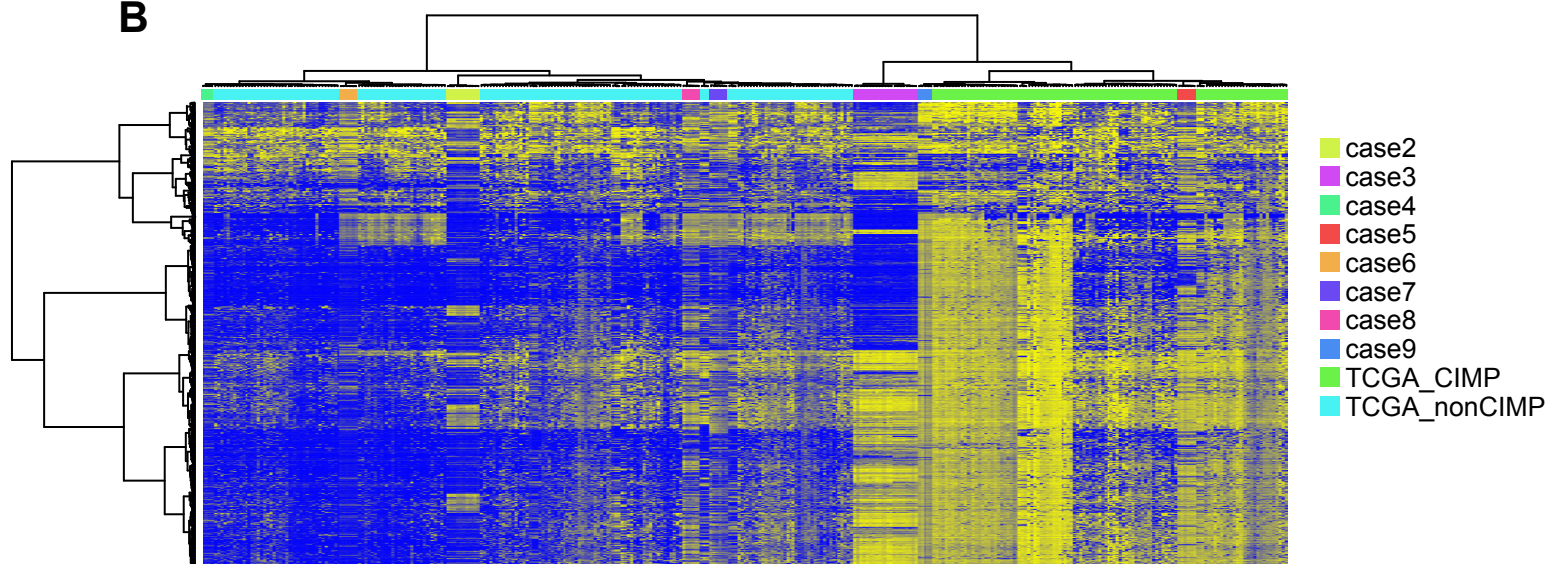

Supplement: S11 Fig — Methylation datasets of 529 TCGA COADREAD samples were obtained from https://tcga-data.nci.nih.gov/tcga/. For the 2000 probes showing the highest variance in the TCGA samples, we made clustered heat maps of β values for only the TCGA samples (A) and the TCGA samples mixed with our samples (B). Based on this result, we classified cases 5 and 9 into the CIMP subtype. (PDF) [file pgen.1005778.s011.pdf]

**A**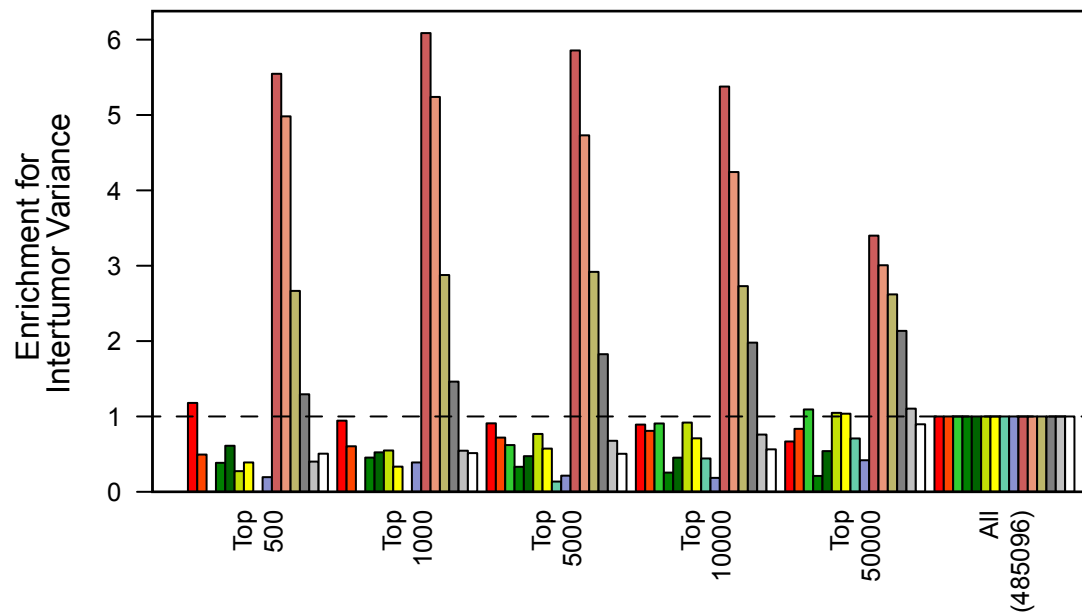**B**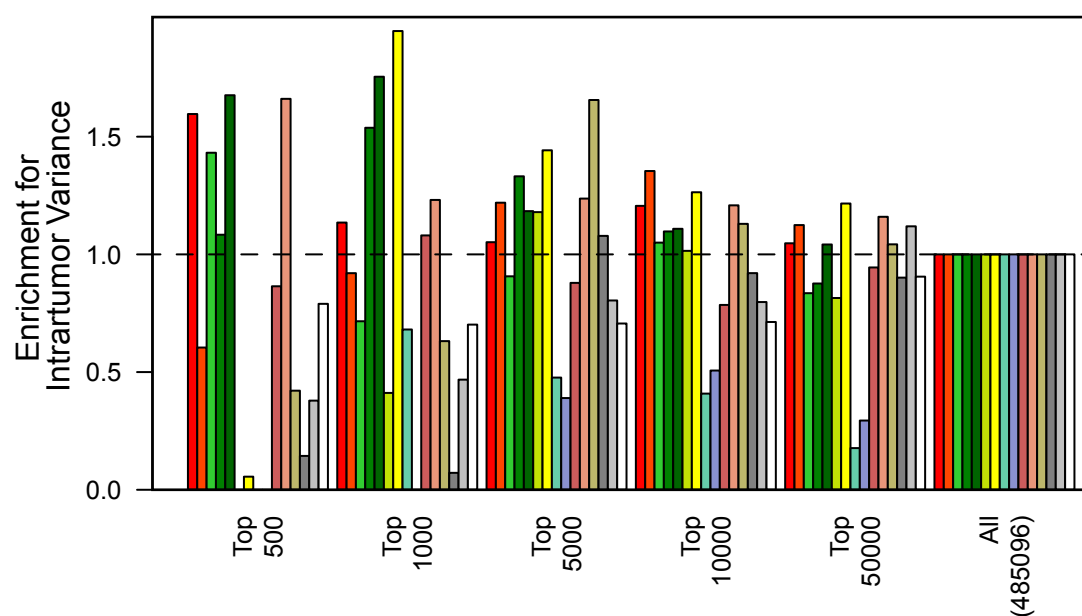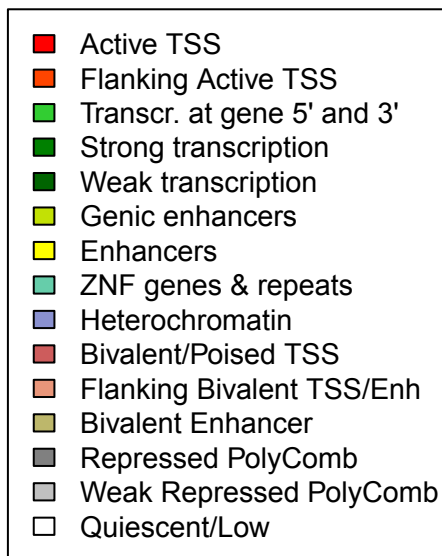

Supplement: S12 Fig — Enrichment scores for intratumor (A) and intertumor (B) variance were calculated as in Fig 4B. Classification of chromosomal regions was based epigenetic status in normal colon tissue, which is profiled by the NIH Roadmap Epigenomics Consortium [13]. (PDF) [file pgen.1005778.s012.pdf]

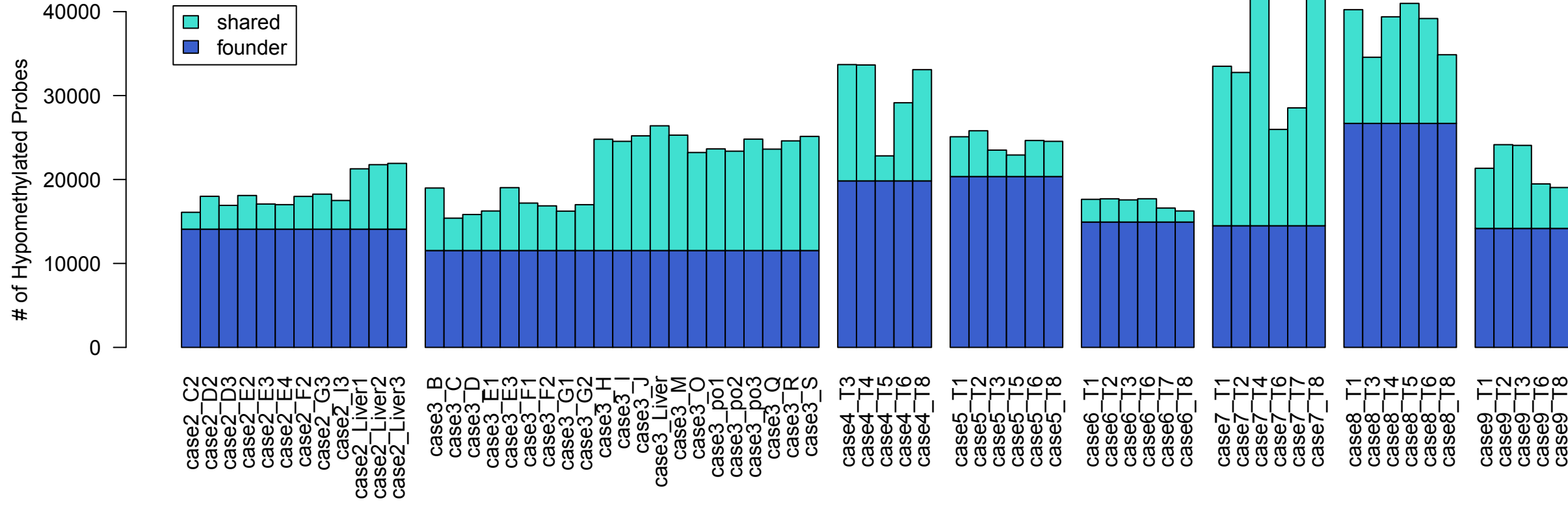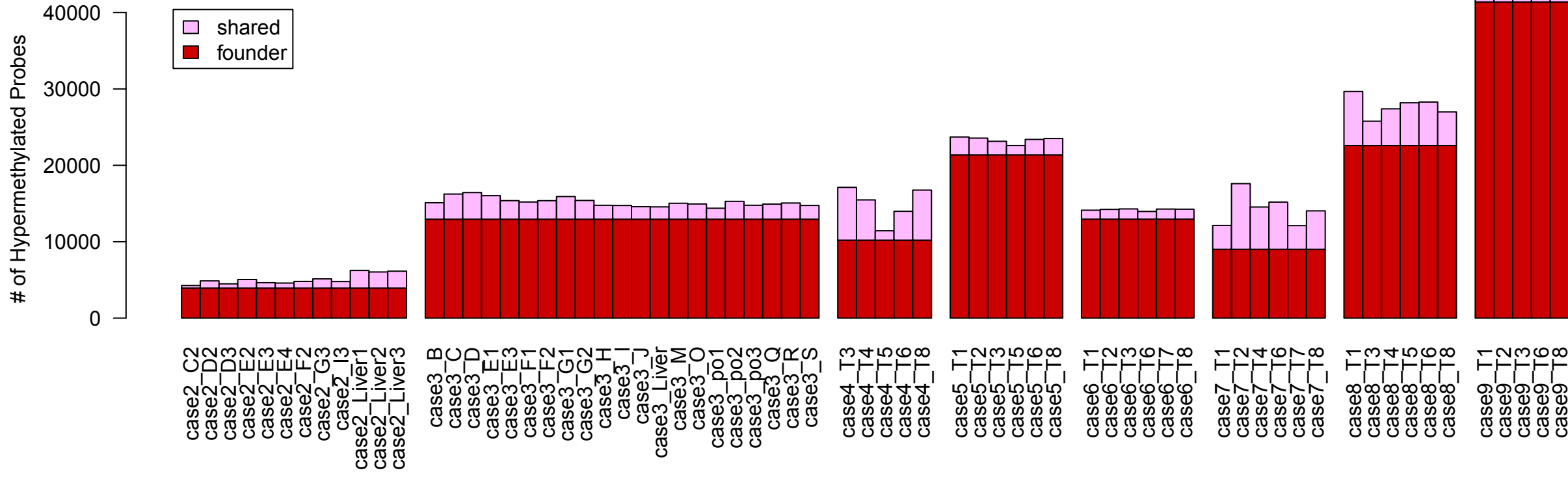

Supplement: S13 Fig — (PDF) [file pgen.1005778.s013.pdf]

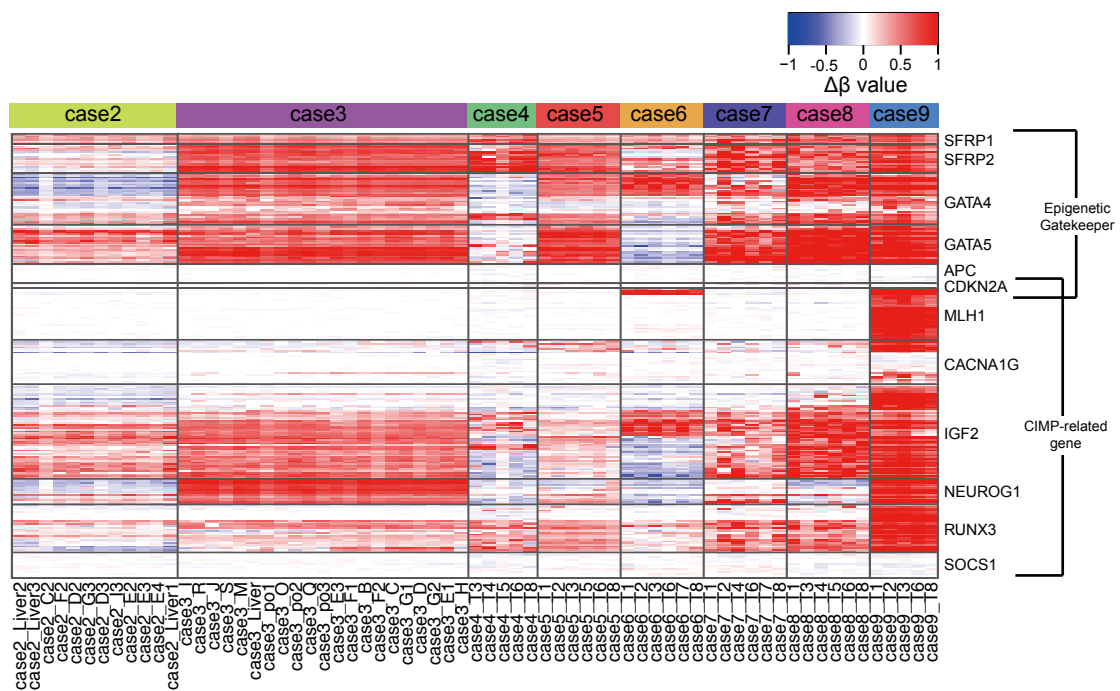

Supplement: S14 Fig — The heat map shows Δβ values of probes contained by the CpG island promoters of the epigenetic gatekeepers [14] and CIMP-related genes [39]. (PDF) [file pgen.1005778.s014.pdf]

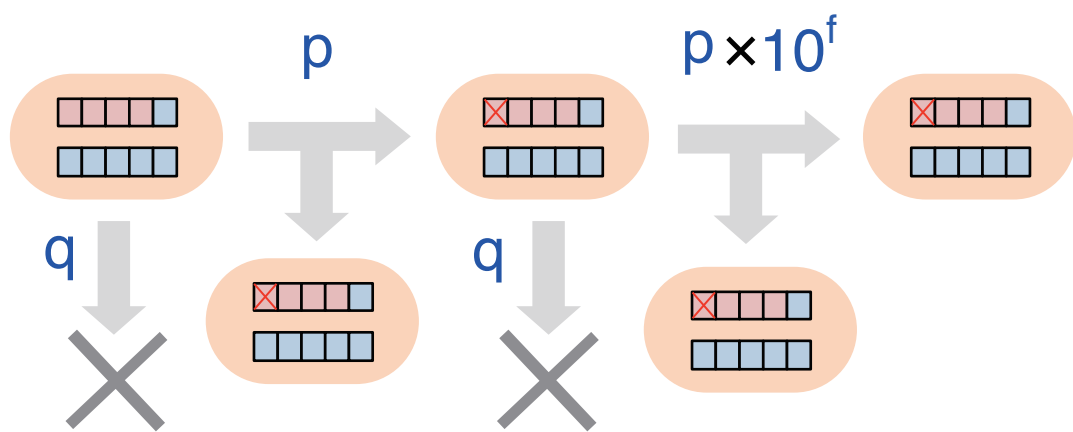

Supplement: S16 Fig — A cell has n genes, d out of which are driver genes. In this schema, n = 10 and d = 4, and red and blue boxes denote driver and non-drive genes, respectively. In a unit time step, a cell divides or dies with probabilities p and q, respectively. During each cell division, each gene is randomly mutated with a probability r, and one driver mutation, which is denoted by a red cross, increases p by 10f -fold. (PDF) [file pgen.1005778.s016.pdf]

**A**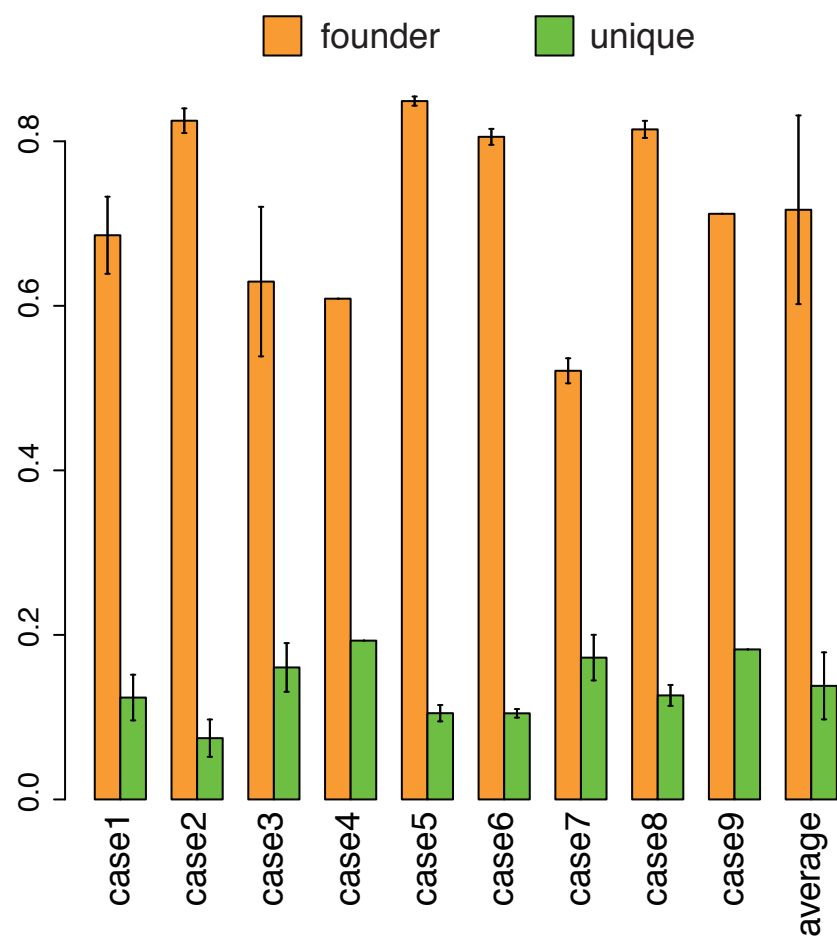**B**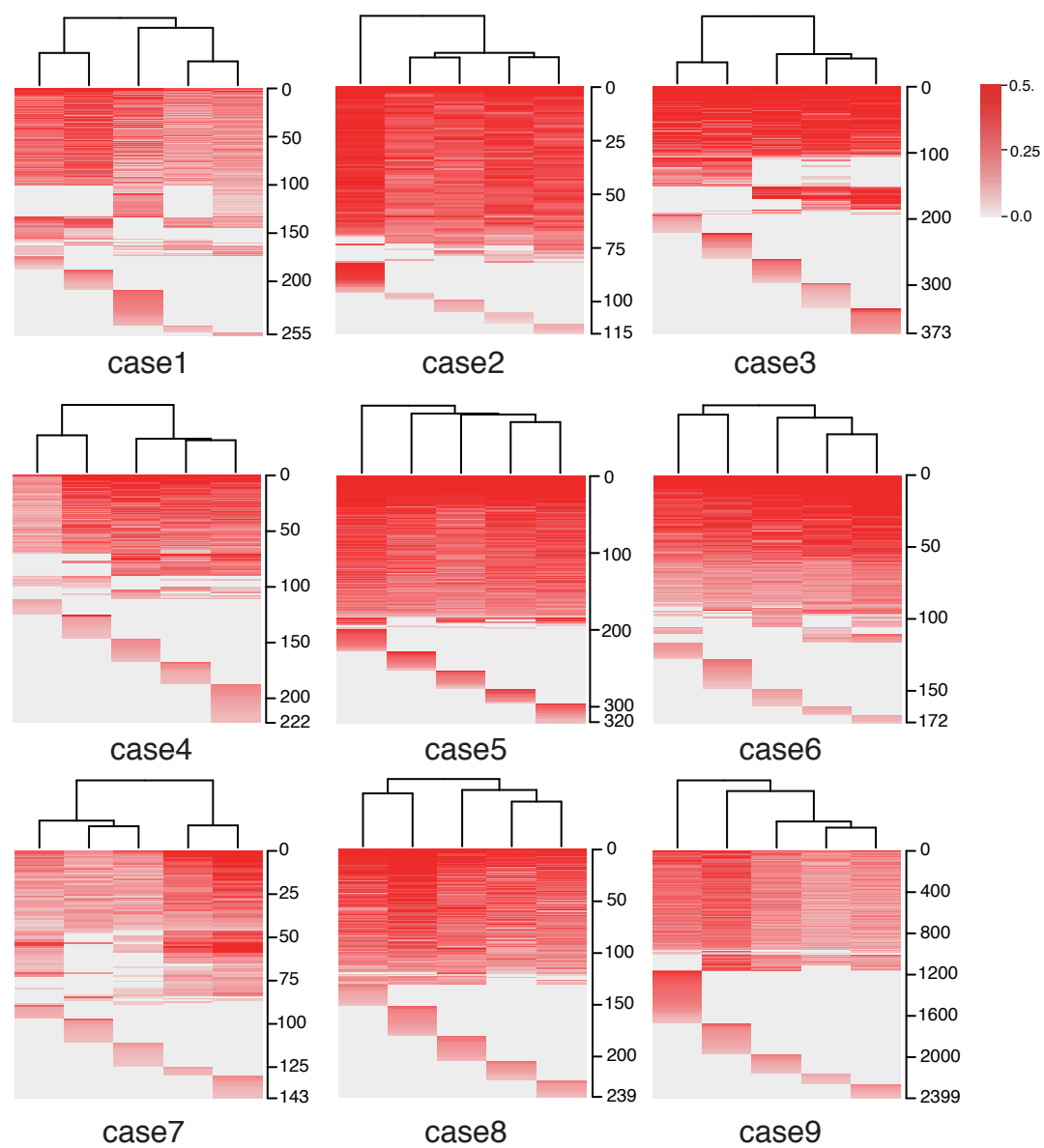

Supplement: S17 Fig — (A) Observed values of summary statistics in the real data. The proportion of founder and unique mutations were obtained for 9 cases. For each of the cases except case4 and case9, downsamplings were performed to obtain 10 multiregional profiles of 5 samples and the statistics were averaged over the downsampling trials. The error bars indicate standard deviations for the downsampling trials. Finally, an “average” over the 9 cases was obtained as an estimate of the observed value of each summary statistic. The error bars at “average” indicate standard deviations over the 9 cases. (B) Multiregional mutation profiles from the real experiments. For the cases except case4 and case9, representative samples from the 10 downsampling trials were presented as in Fig 5C. (PDF) [file pgen.1005778.s017.pdf]

**A**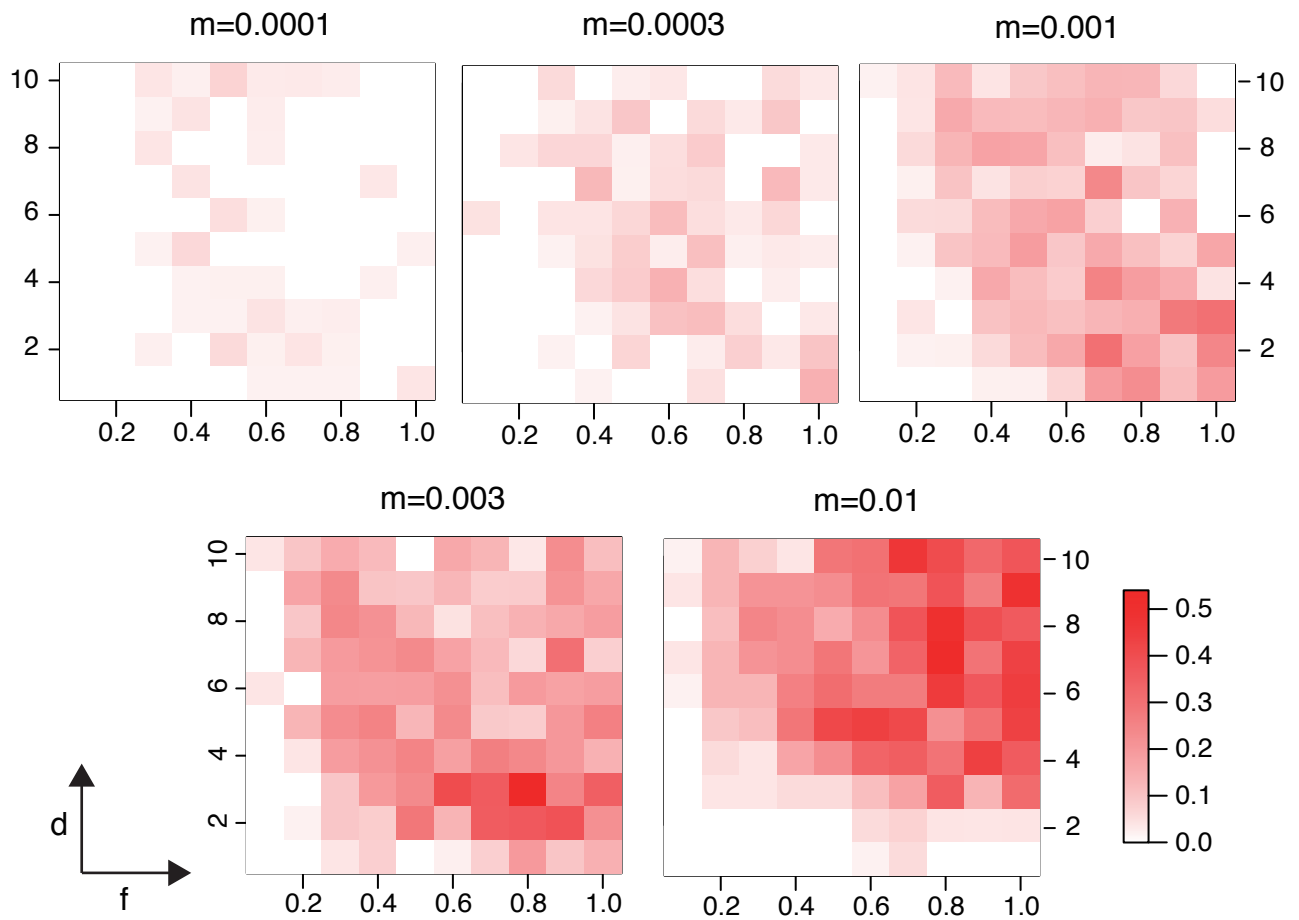**B**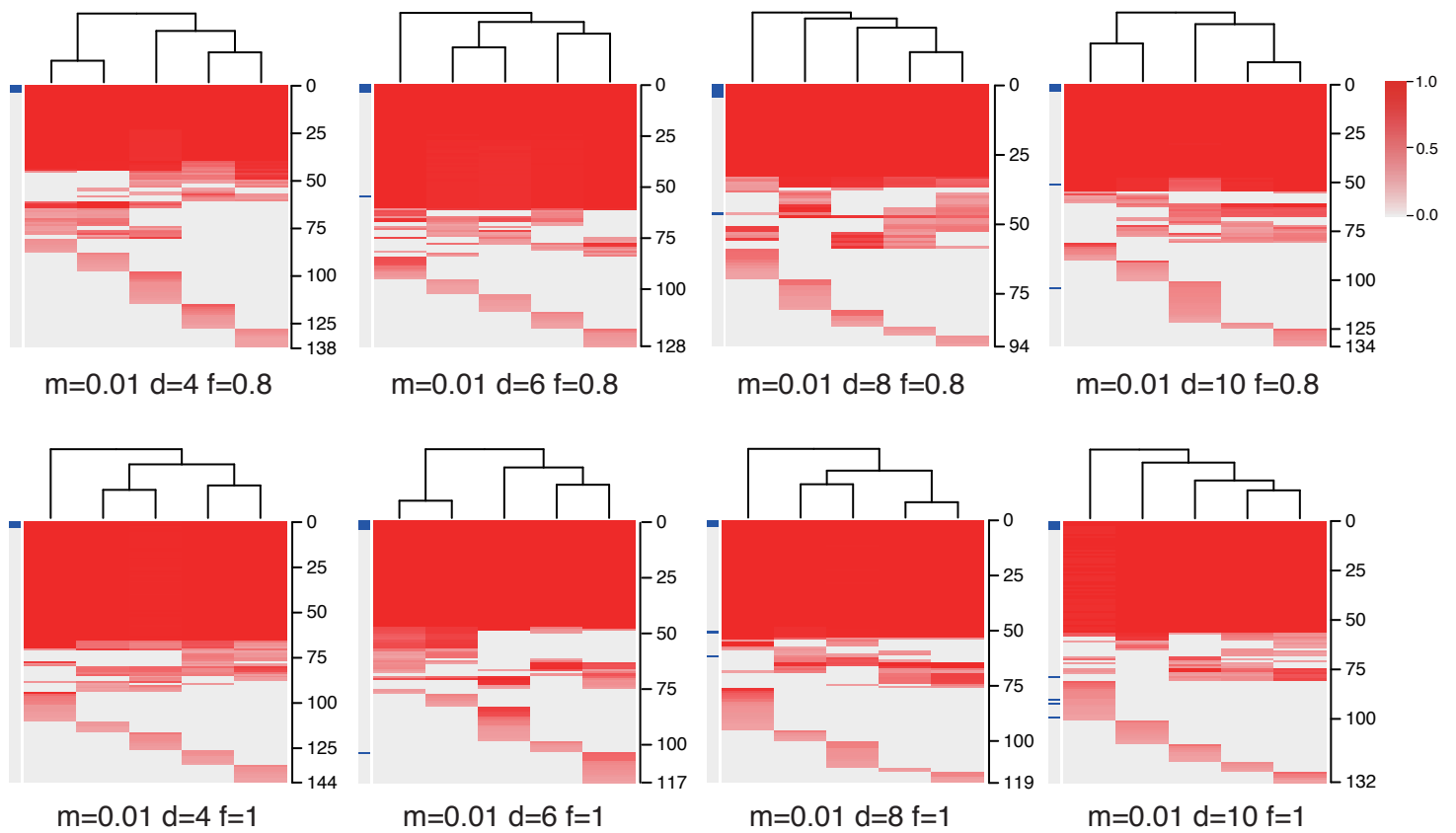

Supplement: S18 Fig — (A) The proportion of simulation instances fitted to the real data. The proportion of simulation instances whose statistics fall within 1 standard deviation from the mean of the observed values was calculated for each parameter settings and visualized as heat maps (B) Multiregional mutation profiles from the simulations. Representative instances from simulation with indicated parameter settings were presented as in Fig 5C. Left blue bars indicate driver genes. (PDF) [file pgen.1005778.s018.pdf]

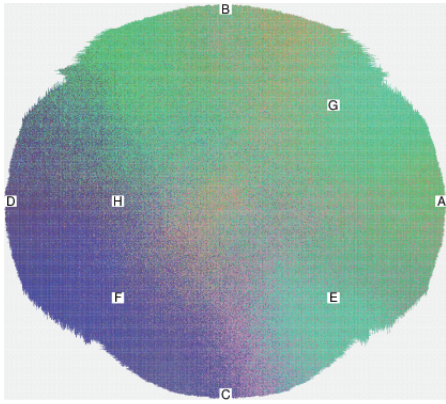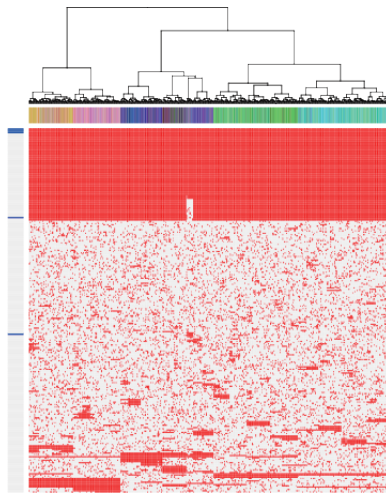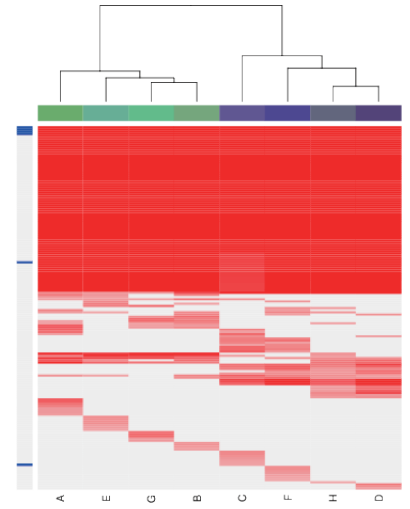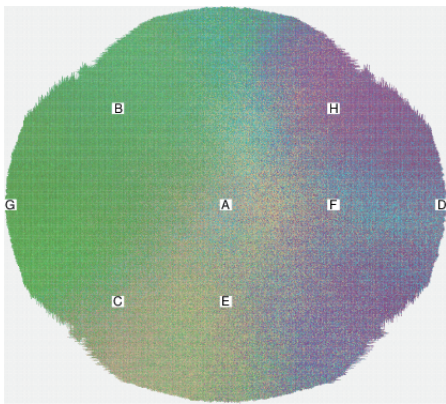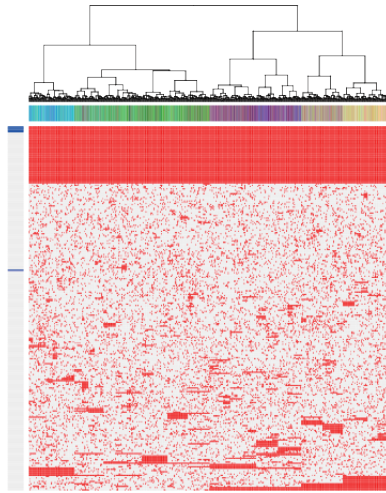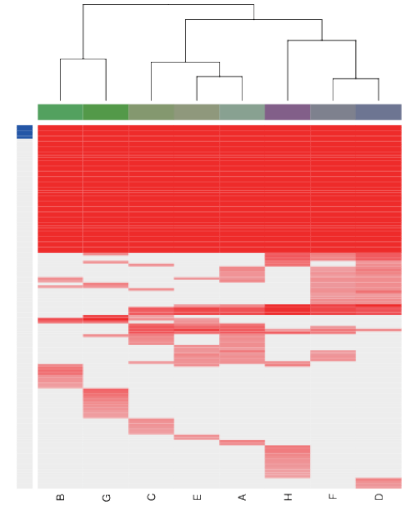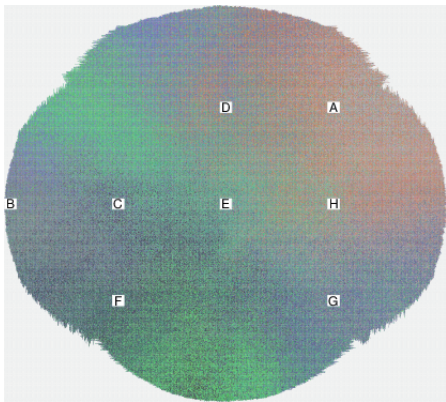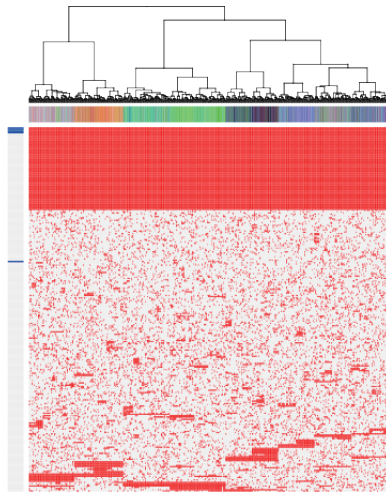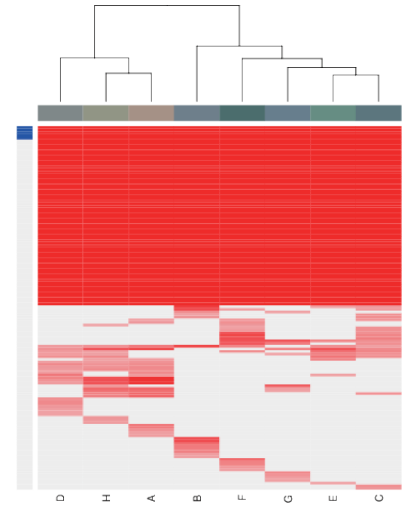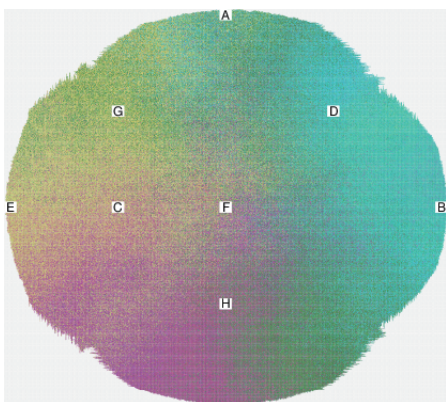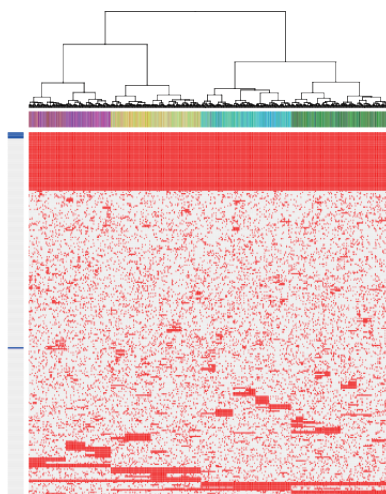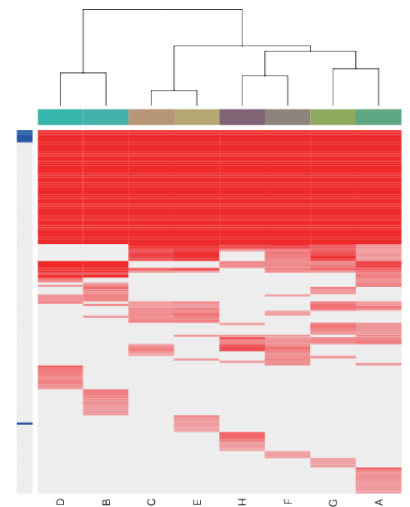

Supplement: S19 Fig — Simulated tumors, simulated single-cell and multiregional mutation profile matrix from four simulation trials are shown as in Fig 5A–5C. (PDF) [file pgen.1005778.s019.pdf]

**A**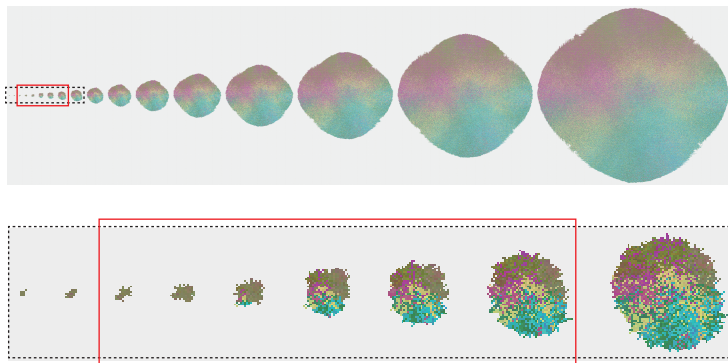**B**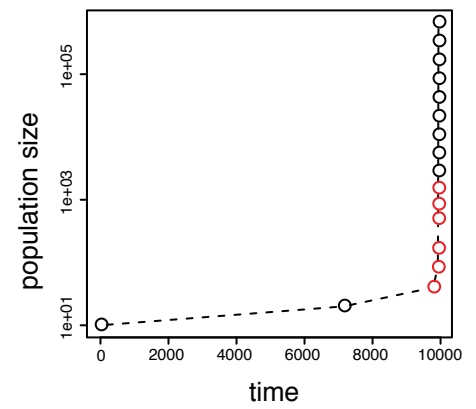**C**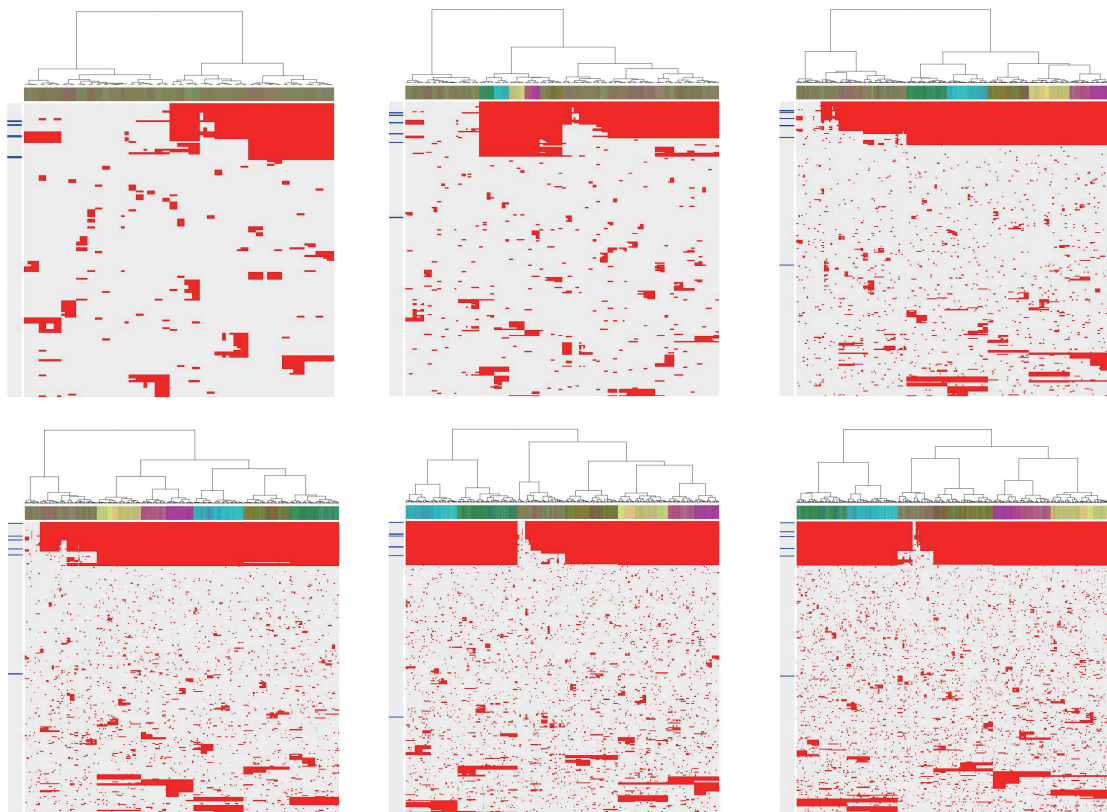

Supplement: S20 Fig — (A) Snap shots of growing tumors in a simulation. Differently colored cell populations represent each clone. (B) A growth curve of the simulated tumor. The snap shots were obtained at each plotted point. (C) Single-cell mutation profiles of the simulated tumor during growth. Timing at which the mutation profiles were obtained are indicated by red rectangles in (A) and red plotted points in (B). Top colored bars represent each clone while left blue bars represent driver genes. (PDF) [file pgen.1005778.s020.pdf]

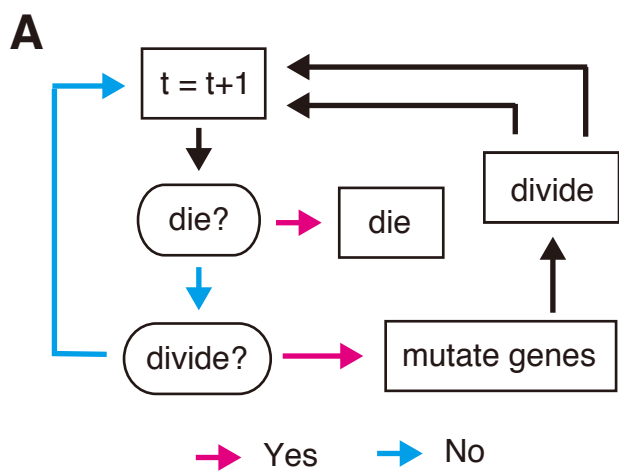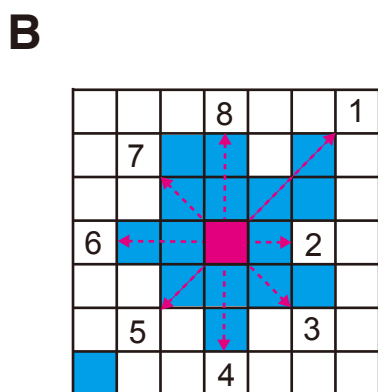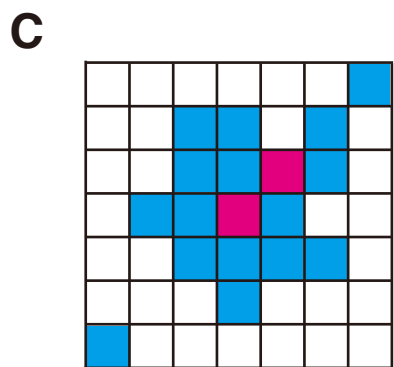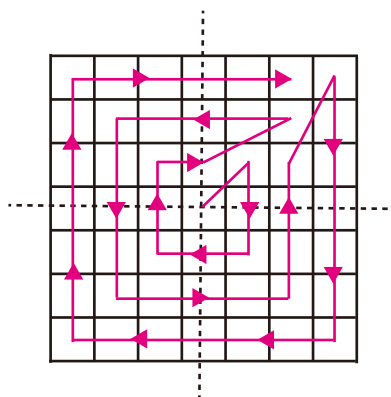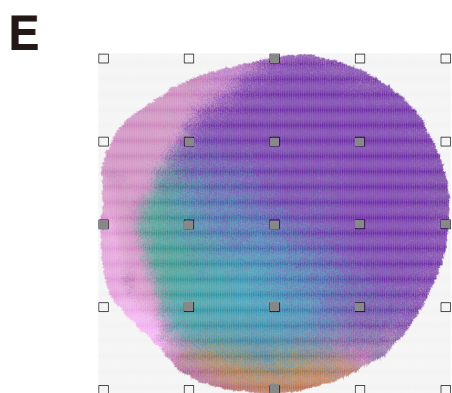

Supplement: S21 Fig — (A) A flowchart of our simulation. (B, C, D, and E,) illustration of division operation. See the simulation section in Materials and Methods. (PDF) [file pgen.1005778.s021.pdf]
