# Supplementary material for: Integrated Multiregional Analysis Proposing a New Model of Colorectal Cancer Evolution
Source: PLoS Genet. 2016 Feb 18;12(2):e1005778. doi: 10.1371/journal.pgen.1005778 (PMC4758664; doi:10.1371/journal.pgen.1005778)
Supplement: S15 Fig — We assumed that hyper- and hypomethylated probes have Δβ> 0.3 and Δβ< -0.3, respectively. CpG island hypermethylation was significantly correlated with patients’ ages. r’s are Pearson’s correlation coefficients and p-values were calculated by the Pearson's correlation test. (PDF) [file pgen.1005778.s015.pdf]

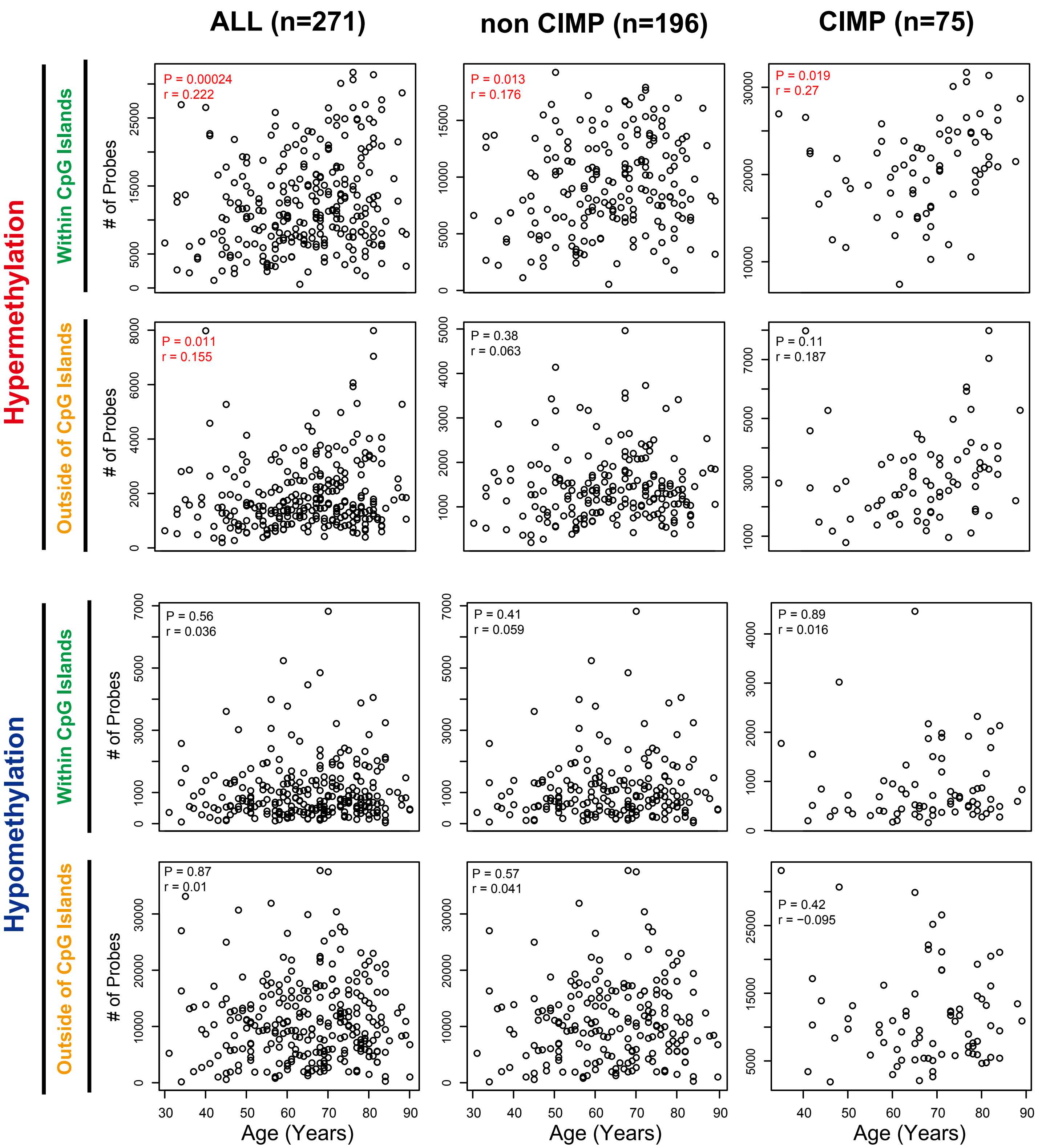

ALL (n=271)
non CIMP (n=196)
CIMP (n=75)
Hypermethylation
Hypomethylation
Within CpG Islands
Outside of CpG Islands
# of Probes
Age (Years)
P = 0.00024
r = 0.222
P = 0.013
r = 0.176
P = 0.019
r = 0.27
P = 0.011
r = 0.155
P = 0.38
r = 0.063
P = 0.11
r = 0.187
P = 0.56
r = 0.036
P = 0.41
r = 0.059
P = 0.89
r = 0.016
P = 0.87
r = 0.01
P = 0.57
r = 0.041
P = 0.42
r = −0.095
